# Influence of Drug Incorporation on the Physico-Chemical Properties of Poly(l-Lactide) Implant Coating Matrices—A Systematic Study

**DOI:** 10.3390/polym13020292

**Published:** 2021-01-18

**Authors:** Daniela Arbeiter, Thomas Reske, Michael Teske, Dalibor Bajer, Volkmar Senz, Klaus-Peter Schmitz, Niels Grabow, Stefan Oschatz

**Affiliations:** 1 Institute for Biomedical Engineering, Rostock University Medical Center, Friedrich-Barnewitz-Straße 4, 18119 Rostock, Germany; michael.teske@uni-rostock.de (M.T.); dalibor.bajer@uni-rostock.de (D.B.); volkmar.senz@uni-rostock.de (V.S.); schmitz@iib-ev.de (K.-P.S.); niels.grabow@uni-rostock.de (N.G.); stefan.oschatz@uni-rostock.de (S.O.); 2 Institute for Implant Technology and Biomaterials e.V., Friedrich-Barnewitz-Straße 4, 18119 Rostock, Germany; thomas.reske@uni-rostock.de

**Keywords:** drug-eluting stent (DES), poly-l-lactide, drug delivery coatings, mechanical properties, thermal properties, surface morphology, paclitaxel, sirolimus, dexamethasone, cyclosporine A

## Abstract

Local drug delivery has become indispensable in biomedical engineering with stents being ideal carrier platforms. While local drug release is superior to systemic administration in many fields, the incorporation of drugs into polymers may influence the physico-chemical properties of said matrix. This is of particular relevance as minimally invasive implantation is frequently accompanied by mechanical stresses on the implant and coating. Thus, drug incorporation into polymers may result in a susceptibility to potentially life-threatening implant failure. We investigated spray-coated poly-l-lactide (PLLA)/drug blends using thermal measurements (DSC) and tensile tests to determine the influence of selected drugs, namely sirolimus, paclitaxel, dexamethasone, and cyclosporine A, on the physico-chemical properties of the polymer. For all drugs and PLLA/drug ratios, an increase in tensile strength was observed. As for sirolimus and dexamethasone, PLLA/drug mixed phase systems were identified by shifted drug melting peaks at 200 °C and 240 °C, respectively, whereas paclitaxel and dexamethasone led to cold crystallization. Cyclosporine A did not affect matrix thermal properties. Altogether, our data provide a contribution towards an understanding of the complex interaction between PLLA and different drugs. Our results hold implications regarding the necessity of target-oriented thermal treatment to ensure the shelf life and performance of stent coatings.

## 1. Introduction

Drug-eluting stents (DES), in particular those of newer generations, are superior to bare metal stents in the treatment of coronary artery stenosis [1,2,3,4,5]. Among others, frequently used drugs are from the Limus family, especially sirolimus (SIR) [6,7,8,9], but also paclitaxel (PTX) [10], dexamethasone (DEX) [11,12], and cyclosporine A (CYCLO) [13,14]. SIR is mainly used for its immunosuppressant effects to inhibit organ transplant rejection, but also shows mammalian target of rapamycin (mTOR) inhibition, reducing cell proliferation and thus the risk of in-stent restenosis [15,16]. PTX is being applied directly to the target area with drug-eluting balloons or in polymer coatings of DES [3,17], however this has recently been questioned [18]. The mechanism of action for PTX is the inhibition of smooth muscle cell (SMC) migration and proliferation by suppression of spindle microtubule assembly during mitosis cycle [16,19]. Dexamethasone (DEX), a corticosteroid, is used for its immunosuppressive and anti-inflammatory properties [20]. Another active pharmaceutical ingredient (API) for potential DES application is CYCLO, which blocks calcineurin-cyclophylin complexation and thus inhibits SMC proliferation and migration [21,22].

Besides polymer-free DES, one of the most common technologies is the drug incorporation into a polymer coating applied to the stent [23]. A wide range of polymers are in clinical use regarding drug carrying matrices for DES [24,25]. Alongside chemically inert polymers, such as fluorinated polymers, polylactide (PLA) and its derivatives are widely used as coating matrices due to the easy processability and their biodegradable nature [26]. Table 1 gives a brief overview about the state-of-the-art of available DES.

In this context, effort has been taken, including work from our group, to understand and modify drug release behavior from polymer/drug matrices. Several reports regarding the release profile of PTX loaded PLA film coatings can be found in the literature [28,29,30,31,32]. Furthermore, in particular SIR loaded DES has been investigated to an extent [33,34,35,36]. Also, release profiles of DEX/PLLA [37] and CYCLO/PLLA [13] are well known from the literature. 

As DES coatings usually possess a thickness in a range of only 10 µm for hemodynamic reasons, they are prone for fracturing or peeling off due to mechanical stress during implantation which is caused by stent insertion and dilatation [4,38]. Therefore, a drug containing coating does not only have to show appropriate release and biocompatibility, but furthermore a suitable mechanical performance paired with resilience against delamination and particle detachment, which is required e.g., in DIN EN ISO 25539-2 [39]. 

For relatively stiff and rigid polymers such as PLLA, blending with other polymers, e.g., PCL, is a frequently used technique to create suitable and sufficient ductile coatings [40,41,42]. In a recent work, we investigated the influence of PCL fraction on the mechanical, morphological and thermal behavior of high molecular weight (HMW) PLLA film materials [43]. Another way to modify the polymer properties is the use of small molecular additives. The fact that the incorporation of such additives into a polymer may lead to plasticizing or hardening effects by intermolecular interactions is well known [44,45,46,47,48]. As for that, Ljungberg and Wesslén reported on the influence of several additives on the mechanical and thermal properties of *PLA*. Triacetine and tributyl citrate proved to be effective plasticizers up to an extent of 25 *w*/*w*% where saturation occurred and the polymer-additive mixture showed phase separation upon heating, accompanied by an increase in PLA crystallinity due to enhanced molecular mobility [49]. 

However, even the incorporation of a drug alone, being a small molecule, the API may lead to the desired increase in flexibility and resilience against fracturing. Regarding the influence on the material properties of drugs incorporated into a polymer matrix, Siepmann, le Brun, and Siepmann reported on acrylic acid copolymers mixed with ibuprofen, chlorpheniramine maleate and metoprolol tartrate [50]. One of their findings was that by the addition of 20 *w*/*w*% ibuprofen, the glass transition temperature (*T_g_*) of the polymer was decreased from 80 °C down to 20 °C accompanied by a strong increase of elongation at break. An innovative concept to overcome such effects, which are not always beneficial or even unwanted, is the encapsulation of the API in a hollow PLA nanoparticles, as reported by Lai’s group [51,52]. Using DSC and IR measurements, they showed that this approach allows a physical separation between the drug and the carrier matrix, so that the drug does not interact with the polymer chains and matrix properties are not altered. Furthermore, due to the diffusion barrier, the encapsulation leads to a decreased burst release. 

All those effects are of certain interest regarding a functional implant, especially in the field of stent devices, which have to be crimped and diluted and are even exposed to intense mechanical stress during the implantation process. Still, only little is known on how the integration of drugs as small molecules into the polymer matrix alters the physico-chemical and mechanical properties of a DDS coating in its entirety.

In the current literature, mostly release behavior of drug containing PLA has been addressed. Although it can be estimated, the influence a drug has on the matrix, such as a shift in *T_g_* or changes in crystallinity, can differ greatly [53,54]. The present study addresses the outcome of the incorporation of selected drugs, SIR, PTX, DEX, and CYCLO, which are approved or under investigation for the local treatment regarding stent related complications, on the mechanical, thermal and morphological properties of PLLA thin films regarding the functionality as DES coatings. 

The drug inclusion criterion for our study was to identify candidates with a high relevance in DES applications, but at the same time representing substantially different chemical base structures. While SIR is a macrocyclic lactone, PTX is a diterpene, DEX possesses a sterol backbone, and CYCLO is a cyclic peptide. Overall, the selected API cover a relevant range of different structural properties in terms of molecular weight, size, polarity, hydrophilicity or functional groups and therefore possible ways of interacting with the polymer matrix. Some indicators for these parameters are polar surface area (PSA) and partition coefficient (logP). Selected characteristics of SIR, PTX, DEX and CYCLO have been summarized in Table 2. To visualize how the drugs used differ in terms of molecular size and shape, structural formulas and space-filling model illustrations were included. 

Our interest was to what extent blending PLLA with different drugs alone may influence or even have beneficial effects on the polymer properties. As shown in Table 1, commercially available DES vary in drug loading. This is both dependent on stent design, as well as on the incorporated API, as different drugs show different therapeutic windows. Thus, literature reports on incorporated drugs for DES technology range from 1 *w*/*w*% for PTX [60] up to 200 *w*/*w*% for DEX [37]. In order to generate comparable data for the selected drugs, we decided to investigate the alteration of polymer properties after incorporation of 10, 15, and 20 *w*/*w*% of drug in PLLA, knowing that certain drug coatings for clinical use may contain lower or higher drug amounts.

It must be noted that the topic of drug incorporation becomes even more complex when considering the effect of additives on polymer/drug matrices. There are several reports in the literature on how the addition of stabilizers or supporting molecules may enhance drug release, mechanical resilience or stability of the carrying matrix on a high level [61,62,63]. However, three or more component systems are a somewhat complex matter in respect to their physico-chemical properties as each part may interact with all the others alone and at once. Consequently, our focus was on binary systems formed by drug incorporation though the authors of this study are aware of the fact that several other compounds are of high interest regarding DES technology. To the best of our knowledge, the influence of commonly used drug molecules on the physico-chemical properties of matrix polymers, such as PLLA, against the background of DES coatings has not been systematically investigated. For this work, as one of the most widely used coating technique [64], especially in industrial application, drug loaded PLLA films were generated via airbrush spray-coating process.

In summary, our aim in the presented study was to analyze the effects of different drug candidates on the properties of the respective polymer/drug matrices from a biomedical engineering viewpoint. It addresses thermal and mechanical properties, long term stability, and phase behavior of PLLA/drug blends, which are parameters of crucial relevance to ensure shelf life and performance of stent coatings. In addition to the already comprehensive literature data concerning drug release behavior and biocompatibility, our results shall provide a complementary contribution towards an understanding of such drug coatings.

## 2. Materials and Methods 

### 2.1. Chemicals

Chloroform and methanol were received from VWR international (Darmstadt, Germany) in technical grade quality. 

HMW PLLA used was Resomer L 210 S, intrinsic viscosity in chloroform: 3.8 dL/g, M_w_ = 320 kDa (Evonik Industries AG, Essen, Germany). Drugs used were: Paclitaxel (Cfm Oskar Tropitzsch GmbH, Marktredwitz, Germany), sirolimus (Cfm Oskar Tropitzsch GmbH, Marktredwitz, Germany), cyclosporine A (Synopharm GmbH & Co. KG, Glinde, Germany) and dexamethasone (Dr. Gerhard Mann Chem.-Pharm. Fabrik GmbH, Berlin, Germany).

All chemicals were used as received without further purification.

### 2.2. Preparation of Polymer Films via Spray Coating

Thin film specimens of PLLA with incorporated drugs were produced using airbrush spray coating process as was reported by our group before [35]. In brief, PLLA was dissolved in CHCl_3_ and drug solution in MeOH (PTX, SIR, DEX) or CHCl_3_ (CYCLO) was added. For each drug, a final ratio of 10 *w*/*w*%, 15 *w*/*w*% and 20 *w*/*w*% was adjusted with respect to the dissolved polymer. As reference, pure PLLA film was manufactured in the same manner without the addition of drugs.

Following to this, thin films of thicknesses of around 7 µm were generated using a custom-made airbrush device on rectangular glass slides as substrate (6.5 × 2.5 cm^2^). Residual solvent from the process was removed from the coated glass slides under reduced pressure at 80 °C. A total amount of approximately 250 µg polymer film was formed and checked via weighing. Films were removed manually from the glass substrates using a scalpel to slightly lift the films, which led to easy detachment.

### 2.3. Raman Microscopy Imaging

Polymer films obtained via spray coating were investigated with regard to drug distrubution at the surface by means of Raman imaging. A WITec alpha 300 confocal Raman microscope (WITec GmbH, Ulm, Germany) with 10× magnification equipped with an input laser with a wavelength of ν = 532 nm was used. For Raman area scans, a 500 µm × 500 µm field with 50 dots per row and column was used, giving a resulting resolution of 10 µm^2^. The area ratio of signals that are specific for the PLLA matrix ($\tilde{v}\;$= 878 rel cm^−1^) and the distinct drugs (PTX: $\tilde{v}\;$= 1012 rel cm^−1^, SIR: $\tilde{v}\;$= 1642 rel cm^−1^, DEX: $\tilde{v}\;$= 1668 rel cm^−1^) were chosen for visualizing drug distribution. The areas of distinct drug signals identified from preliminary measured reference spectra have been normalized to the area of the PLLA signal at $\tilde{v}\;$= 883 rel cm^−1^. The measurements have been performed for two separately manufactured drug containing PLLA films and the median of the quotients was formed. In the case of CYCLO incorporation, no distinct drug signal could be detected. 

### 2.4. FTIR Measurements

**Figure 1:**
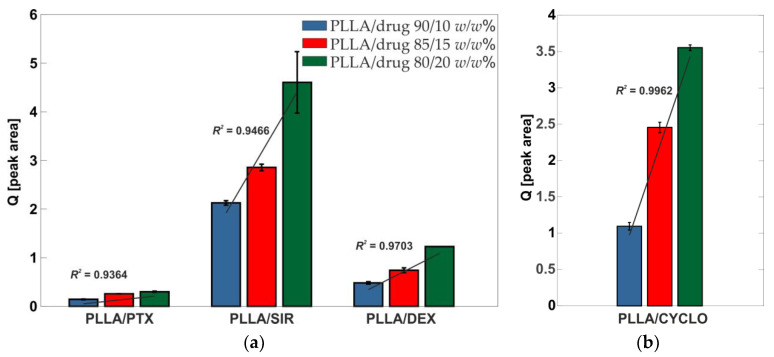
(**a**) Quotients of drug signal found in Raman spectroscopy (PTX: $\tilde{v}\;$ = 1012 rel cm^−1^, SIR: $\tilde{v}\;$ = 1642 rel cm^−1^, DEX: $\tilde{v}\;$ = 1668 rel cm^−1^) compared to PLLA reference signal to ensure drug loading. (**b**) Quotient of the CYCLO signal at $\tilde{v}\;$ = 1629 cm^−1^ from FT-IR spectroscopy compared to PLLA reference signal. Linear regression of the means has been performed and *R*^2^ values are given.

FTIR-ATR-measurements were performed using a Bruker Vertex 70 IR-Spectrometer (Bruker, Leipzig, Germany) equipped with a DLaTGS-detector. Data were collected in the range of $\tilde{v}\;$= 500 cm^−1^ to 4000 cm^−1^ with a resolution of $\tilde{v}\;$= 4 cm^−1^ averaged over 100 scans in reflection mode using a Graseby Golden Gate Diamond ATR-unit. All spectra were subsequently baseline corrected and atmospheric compensation has been performed. Subsequently, the area of the CYCLO signal at $\tilde{v}\;$= 1629 cm^−1^ has been compared to the area of PLLA signal at $\tilde{v}\;$= 1749 cm^−1^. The quotient formed from these areas in a similar manner as for Raman measurements can be found in Figure 1b.

### 2.5. SEM Imaging

Morphology of the polymer films was examined with scanning electron microscopy SEM QUANTA FEG 250 (FEI Company, Dreieich, Germany) operating in high vacuum and 10 kV, using an Everhart-Thornley secondary electron detector (ETD). The samples were fixed onto aluminum carriers using conductive carbon pads and Au sputter coated. The images were taken at magnification ×1000.

### 2.6. Contact Angle Measurements

Contact angle measurements have been performed using a mobile surface analyzer MSA (Krüss GmbH, Hamburg, Germany) equipped with a double pressure dosing unit at ambient conditions with 2 µL drop volume and 1 s equilibration time. As test liquids, deionized water and diiodomethane were used to calculate free surface energy. Contact angles have been determined in triplicate for each drug loaded polymer film using separate cutouts. Contact angles were calculated by averaging the values for both sides of the drops. To avoid bending of the test samples and to ensure a plain shape, the PLLA films have been mounted on pyrolytic graphite planchets.

### 2.7. Thermal Analysis

The DSC 1 system (Mettler-Toledo, Greifensee, Switzerland) was used to determine the thermal properties of drug-loaded PLLA spray-coated films using the conventional calibration methods with highly pure standards. The specimens were heated up from 25 °C at a rate of 10 K/min operating under nitrogen at atmospheric pressure. The end temperature was between 210 °C and 280 °C, depending on the incorporated drug. The sample weights were in the range of 0.3–2.5 mg. Samples were analyzed with respect to glass transition (*T_g_*), crystallization temperature (*T_c_*), melting temperature (*T_m_*), and degree of crystallinity of PLLA (*χ*). The heats of fusion Δ*H* and crystallization *χ* were quantitatively evaluated by means of Equation (1)

(1) $$\chi =100\%\cdot \left[\frac{\Delta H_{m}}{\Delta H_{m0}}\right]\cdot \frac{1}{W}$$

where Δ*H_m_*_0_ is the enthalpy value of a pure crystalline material, Δ*H_m_* is the enthalpy corresponding to the fusion process and W the amount of each component in the polymer/drug system. The reference value taken for Δ*H_m_*_0_ of PLLA was *χ*_100_ = 93.7 J/g [65]. All results were averaged over n = 5 samples.

### 2.8. Mechanical Testsing

Tensile tests were carried out using the uniaxial tensile test instrument Zwicki ZN 2.5 (Zwick/Roell, Ulm, Germany). Samples were cut into dumbbell shape specimens with effective dimensions of 12 mm × 2 mm according to DIN EN ISO 527-2 1BB standards [66]. Sample thickness was measured using SEM QUANTA FEG 250 (FEI Company, Dreieich, Germany). It was determined using the reverse form of the dogbone sample. 10 mm samples directly neighboring the cutouts of the specimens for mechanical testing were mounted on the edge of the aluminum trays. The carrier was positioned vertically using a 90° adapter in the SEM to obtain a top view of the cutting edge. For each sample, at three distinct positions with n = 3, thickness values were determined. 

Mechanical tests were conducted with a 10 N load cell and a crosshead speed of 25 mm/min. All tests were performed at room temperature (20 °C). The tensile force as a function of elongation was measured. Based on these data, the elastic modulus (*E*) was calculated in the linear elastic region. Furthermore, the tensile strength (*σ_max_*) and elongation at break (*ε_B_*) were determined for n = 5 samples. For statistical analysis, a twofold Nalimov’s test has been performed and data were corrected for outliers.

### 2.9. Statistical Analysis

All data are given as mean ± standard deviation. For statistical analysis of contact angle, mechanical and DSC data, two tailed *t*-tests of the means versus PLLA reference have been performed. For statistical analysis, SigmaPlot software (V13.0, Systat Software, Inc., San Jose, CA, USA) has been used. Significances are given at a significance level of *p* < 0.05 and marked with an asterisk. 

## 3. Results

### 3.1. Spectroscopical Analysis of Drug Loaded PLLA Films

Our first interest was whether drugs were homogeneous distributed into the PLLA films. Therefore, drug incorporation has been ensured by the use of Raman spectroscopy. The resulting quotient $Q=\left[\frac{A_{Drug}}{A_{PLLA}}\right]$ is given in Figure 1a. Raman spectra are given in the supporting information (Supplementary Figure S1).

From Raman data, a linear progression can be seen for all drugs leading to the assumption that no separation and precipitation from the solution during airbrush process occurred and that all drugs are distributed homogeneously in the polymer films. For CYCLO loaded PLLA films, no distinct Raman signal for the drug could be detected. To ensure the successful drug loading, FT-IR spectroscopy has been used. As for the drugs analyzed with Raman, linearity in the drug distribution was observed. 

**Figure 2:**
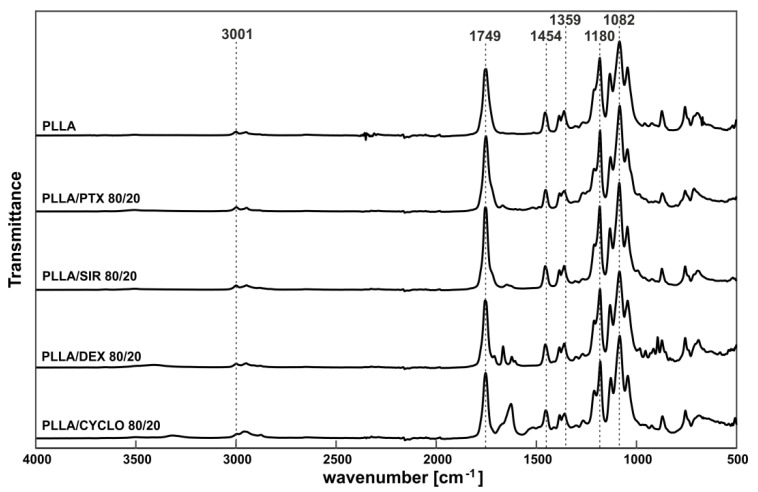
IR spectra of PLLA film and PLLA films with 20 *w*/*w*% incorporated drugs. IR bands of PLLA have been identified with reference to [51].

FTIR-ATR-measurements have been performed for all drug loaded PLLA films to investigate a potential polymer/drug interaction on a molecular level. Figure 2 shows IR spectra for 20 *w*/*w*% drug in PLLA, spectra for 10 *w*/*w*% and 15 *w*/*w*% can be found in the supporting information (Supplementary Figure S2). Characteristic IR bands of PLLA have been assigned according to [51] and are $\tilde{v}$ = 3001 cm^−1^ (CH stretch), $\tilde{v}$ = 1749 cm^−1^ (C=O stretch), $\tilde{v}$ = 1454 cm^−1^ (CH_3_ bend), $\tilde{v}$ = 1359 cm^−1^ (C-H deformation), $\tilde{v}$ = 1180 cm^−1^ (C-O-C stretch), and $\tilde{v}$ = 1082 cm^−1^ (C-O-C stretch).

### 3.2. Influence of Drug Incorporation on the Surface Morphology and Drug Distribution

**Figure 3:**
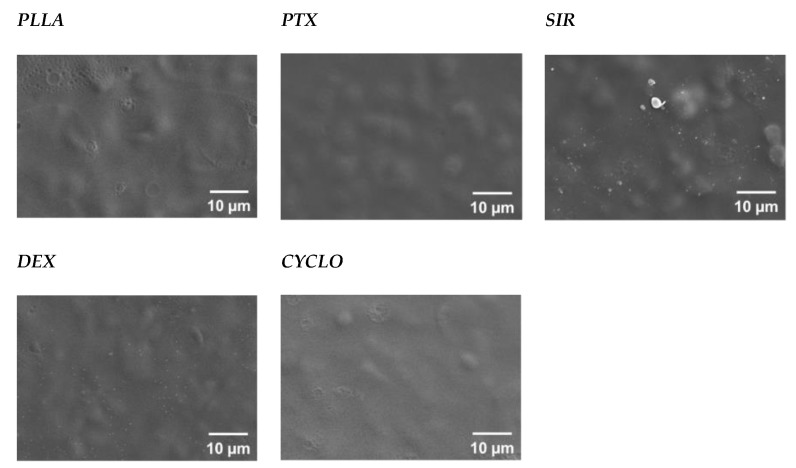
Exemplary SEM images for different drugs incorporated in PLLA samples (PLLA/drug 80/20 *w*/*w*%). Drugs used were paclitaxel (PTX), sirolimus (SIR), dexamethasone (DEX) and cislosporine (CYCLO).

Changes in morphology due to the drug incorporation were investigated by means of macrophotography, scanning electron microscopy and Raman imaging. Image analysis of macro photography for drug containing PLLA films showed no significant changes compared to pure PLLA reference films such as phase separation or roughening. Macroscopic images are given in SI (Supplementary Figure S3). For a more detailed view, SEM imaging has been used to investigate the microscopic appearance of the drug loaded polymer films. At a magnification of 1000×, no such thing as microcrystals or other inhomogenities as well as changes in surface morphology could be observed for PTX and CYCLO. These drug containing films appeared smooth and comparable to the PLLA reference. The crater like shapes are most probably due to solvent evaporation. Regarding SIR and DEX, the surfaces show bumps in submicron range for all investigated concentrations, 10 *w*/*w*%, 15 *w*/*w*% and 20 *w*/*w*%. It must be noted that the side which was attached to the glass slid showed in all cases a plainer appearance. In Figure 3, exemplary SEM images for all PLLA/drug combinations at the highest drug concentration of 20 *w*/*w*% at 1000× magnification are shown. SEM images for 10 *w*/*w*% and 15 *w*/*w*% can be found in Supplementary Figure S4.

**Figure 4:**
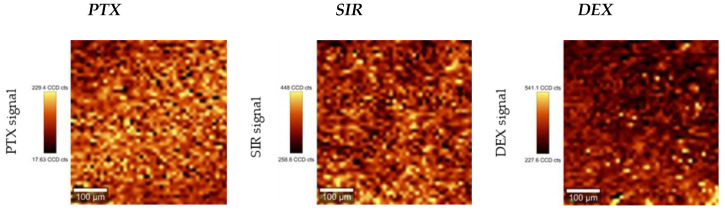
Exemplary Raman mapping images for different drugs incorporated in PLLA (PLLA/drug 20/80 *w*/*w*%). For CYCLO no distinct Raman signal could be detected.

As for Raman microscopy, distinct drug signals (PTX: $\tilde{v}$= 1012 rel cm^−1^, SIR: $\tilde{v}$ = 1642 rel cm^−1^, DEX: $\tilde{v}$= 1668 rel cm^−1^, Supplementary Figure S1) were mapped in respect to their intensity. As mentioned before, CYCLO gave no measurable Raman signal when blended with PLLA and could therefore not be analyzed via Raman mapping. All drugs showed homogeneous distribution even at highest concentration of 20 *w*/*w*%, see Figure 4. No such things as macroscopic crystals, agglomerates or drug enriched phases were observed. Raman mapping images for 10 *w*/*w*% and 15 *w*/*w*% can be found in Supplementary Figure S5.

### 3.3. Influence of Drug Incorporation on Surface Hydrophilicity

**Figure 5:**
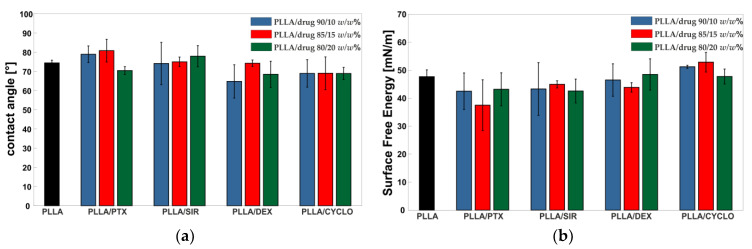
(**a**) Water contact angle and (**b**) surface free energy data for drug incorporated PLLA films (mean values for n = 3 with droplet angles taken each from both sides of the drops are shown). Statistical analysis (two-tailed *t*-test) did not show any significant differences (*p* < 0.05) of contact angle values and SFE values of drug incorporated PLLA films when compared to pure PLLA reference.

Figure 5 gives an overview on the contact angles for the polymer films addressed in this study. Exemplary images of the drops can be found in the Supplementary Figure S6. Compared to PLLA reference film, drug incorporation showed to have no effect on the surface wettability as all contact angles are in a range of 70–80°. Furthermore, surface free energy (SFE) calculated from contact angle data acquired from water and diiodomethane have been calculated. As can be seen, incorporation of the chosen drugs did not result in observable changes in SFE. 

### 3.4. Influence of Drug Incorporation on the Thermal Properties—DSC

**Figure 6:**
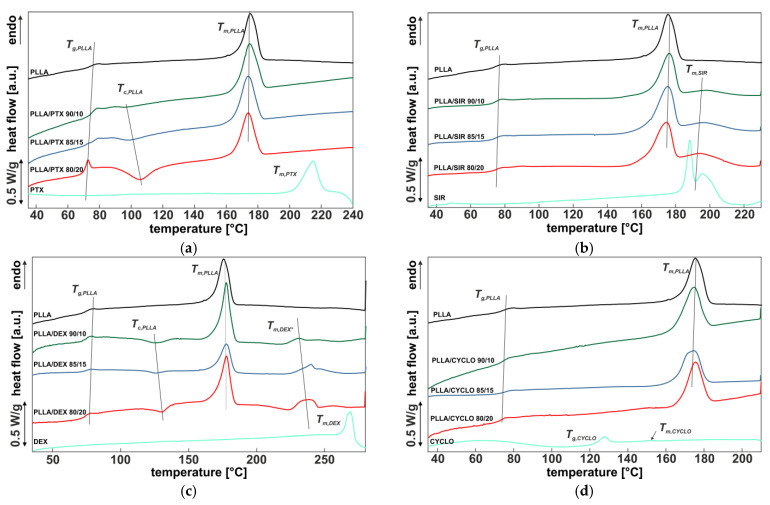
DSC thermograms of PLLA (black), drug (cyan) and PLLA/drug (**a**) PLLA/PTX, (**b**) PLLA/SIR, (**c**) PLLA/DEX and (**d**) PLLA/CYCLO) in different ratios (90/10, 85/15, 80/20 *w*/*w*%). The straight lines are guides for the eyes, only.

Figure 6 shows the first DSC heating curve of the spray coated films with the different drug ratios (90/10, 85/15, 80/20 *w*/*w*%) after thermal annealing. For reference, both the DSC curves of the pure polymer PLLA and the pure drug are included for each diagram.

The DSC heating curves of pure PLLA spray coated film exhibit a glass transition temperature at *T_g_* = 72.8 °C. No exothermic peak, but an endothermic melting peak at 179 °C is observed, which is comparable to reported *T_m_* data for HMW PLLA [67]. For 20 *w*/*w*% PTX, *T_g_* of PLLA is shifted to lower temperatures, as shown in Table 3. Furthermore, an exothermic peak *T_c,PLLA_* is formed with increasing drug content, whose peak temperature is shifted to higher temperatures as the ratio increases (Figure 6a). The melting peak *T_m,PLLA_* is nearly identical at all concentrations. No other endothermic peak was identified that would be indicative of the presence of a pure PTX phase.

SIR incorporation into PLLA (Figure 6b) did not lead to distinct changes in *T_g,PLLA_* compared to pure PLLA. Even the endothermic melting peak *T_m,PLLA_* is kept constant. In addition, a further endothermic peak *T_m,SIR_* at 184 °C is observed, which can be related to the drug according to the reference measuring curve of the pure SIR material. There is no cold crystallization observed as in PTX.

PLLA/DEX DSC curves show no distinct shift in *T_g,PLLA_* (Table 3), but an exothermic peak *T_c,PLLA_* is formed with increasing drug content, whose peak temperature is shifted to higher temperatures as the drug content increases (Figure 6c). The PLLA melting peak *T_m,PLLA_* is almost identical at all concentrations. In addition, there is a further endothermic peak *T_m,DEX_* which can be identified from the measurement curve of the pure DEX material and which is shifted to higher temperatures and broadens as the drug ratio increases.

Thermograms of PLLA including CYCLO show no significant changes compared to pure PLLA (Figure 6d). As for that we conclude complete incorporation of the drug into the polymer matrix. However, as only little to no changes in *T_g,PLLA_* or *T_m,PLLA_* occur, low interaction of the polymer chains with the drug molecules can be assumed. 

**Figure 7:**
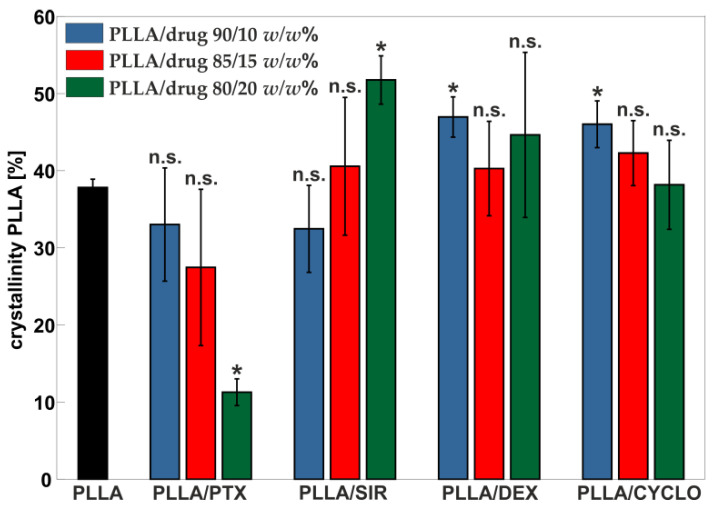
Crystallinity of PLLA in PLLA/drug spray coated films with different ratios (90/10, 85/15, 80/20 *w*/*w*%), determined from DSC measurements. Asterisks (*) mark significant differences of crystallinity data with *p* < 0.05 obtained by two-tailed *t*-test, each in comparison to PLLA reference. n.s. stands for not significant.

To evaluate the influence of drug incorporation on PLLA crystallinity, the percentage values as determined according to Equation (1) are plotted in Figure 7. The black bar refers to PLLA without drug content and with a value of *χ* = 37.8 ± 1.9 %, which is comparable with the literature for HMW PLLA [67]. With increasing PTX content, *χ* values of PLLA decreased from *χ* = 33 ± 7% down to *χ* = 11.3 ± 1.7%. In contrast, *χ* values of PLLA with SIR increased with increasing drug content. PLLA crystallinity including DEX or CYCLO show only little to no changes compared to pure PLLA. All *χ* values are given in Table 3. 

### 3.5. Coating Thickness Determination

For the calculation of mechanical parameters, adequately accurate thickness determination is necessary. A change in thickness accuracy of approximately ±1 µm would result in falsifying elastic modulus and tensile strength values of about 20 %. Consequently, as the films showed thicknesses in a range below 10 µm, thickness determination by the use of a dial indicator was not suitable. 

**Figure 8:**
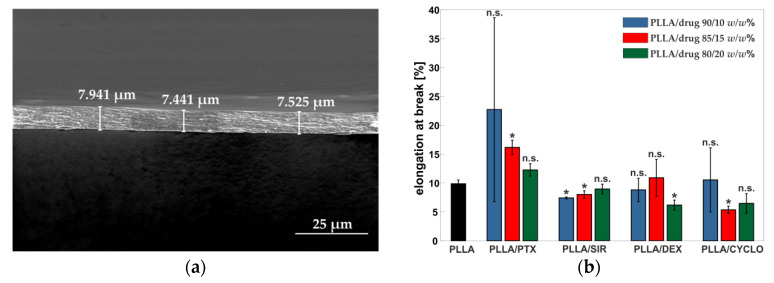

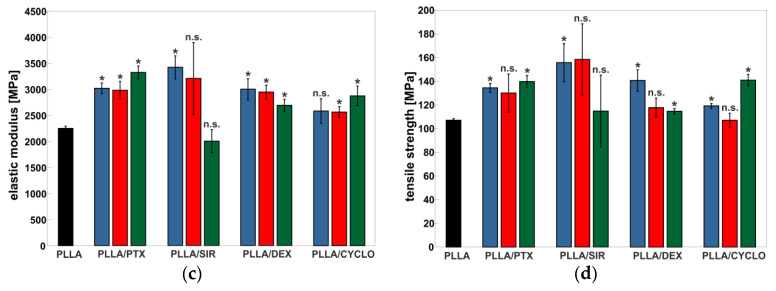
(**a**) Exemplary SEM image of PLLA reference film with determined thicknesses. (**b**–**d**) Mechanical properties ((**b**) elongation at break, (**c**) elastic modulus and (**d**) tensile strength), determined from uniaxial tensile tests of PLLA/drug spray coated films in different drug ratios (90/10, 85/15 and 80/20 *w*/*w*%). Asterisks (*) mark significant differences of the values with *p* < 0.05 obtained by two-tailed *t*-test, each in comparison to PLLA reference. n.s. stands for not significant.

To overcome this issue, film thickness has been determined using SEM imaging. Using a 90° adapter ensures precise sample characterization up to some few nanometers. However, measuring errors due to misalignment of the sample or manual setting of the scale bar have to be considered. As for that, a cutout close to the point of rupture of the strain samples has been threefold measured at three distinct points by SEM. A sample image for one of such measuring points is given in Figure 8a. 

### 3.6. Influence of Drug Incorporation on the Mechanical Properties

An overview on the mechanical parameters is given in Figure 8b–d. From tensile tests, elongation at break, elastic modulus and tensile strength have been calculated.

In general, drug incorporation results in an increase of elastic modulus up to 3420 ± 220 MPa for PLLA/SIR 90/10 compared to pure PLLA with a value of E = 2250 ± 40 MPa. Only SIR at 20/80 *w*/*w*% resulted in a decrease down to E = 2000 ± 220 MPa. Regarding the mechanical behavior, PTX leads to a high deviation for elongation at break values. SIR did not show to have any influence on the elongation at break. For DEX, a tendency to a decrease in elongation at break can be observed. Results for CYCLO also indicate a slight decrease in ε_B_ from 11 ± 6% (10 *w*/*w*%) to 6.4 ± 1.7% (20 *w*/*w*%) compared to PLLA at 9.9 ± 0.7%. All polymer/drug materials show higher tensile strength values up to 160 ± 30 MPa or comparable ones compared to pure PLLA with *σ_max_* = 107.1 ± 1.3 MPa. Results of mechanical properties are given in Table 4 and the stress strain curves are given in Supplementary Figure S7. 

## 4. Discussion

In this work, drug incorporated PLLA films were manufactured by spray-coating using PLLA/drug solutions, which is also the method of choice in industrial settings [64]. This technique allows the generation of homogeneous polymer coatings on complex structures, such as those represented by scaffolds for cardiovascular intervention in particular. Still, the removal of solvent residues remains crucial, as these, even in trace amounts, may show negative effects on biocompatibility as well as influence the mechanical and thermal properties of the coating. To investigate to what extend drugs influence the polymer matrix, rinsing protocols for solvent removal are unsuitable, as such treatments lead to washout of the drugs. For spray-coated thin films, however, thermal annealing above *T_g_* under reduced pressure has shown to be sufficient to remove solvent residues, making rinsing steps unnecessary [35,68]. Nevertheless, thermal treatment may lead to further altering of the properties of the drug containing films, which must be taken into account.

Our initial interest was in macroscopic film morphology and drug distribution, whereas no macroscopic changes were observed. Raman and IR spectroscopy were used to prove the incorporation of drugs in the polymer films. For all drugs except for CYCLO, which was unmeasurable, Raman mapping showed a homogeneous distribution. Furthermore, contact angle and SFE measurements showed that drug incorporation did not result in significant changes even at a high concentration of 20 *w*/*w*%. This is interesting in a way such that the drugs addressed in this study exhibit very different logP values. Moreover, the observed phase separation and formation of microcrystals for DEX and SIR in accordance to DSC measurements appear to have no influence on surface wettability. This leads to the assumption that wettability of drug coating is not affected by drug addition, but remains comparable to the pure PLLA matrix. 

The IR-spectra showed no distinct changes in IR bands but appear as overlapping spectra of PLLA and the incorporated drugs. As for that, we assume no chemical interaction of SIR, PTX, DEX, or CYCLO with the PLLA matrix as no shift in characteristic signals appears.

Regarding thermal analysis, the drugs showed a different behavior. CYCLO showed little to no influence on the polymer matrix and thus barely any effect on the thermal properties.

As for PTX, a slight shift of *T_g_* of the polymer matrix was observed. In the literature, it has been reported that PTX is miscible with the amorphous domains of PLLA, in consequence softening the polymer matrix, leading to a decrease in *T_g_* [69]. Furthermore, this also leads to the formation of an exothermic peak (*T_c_*)_,_ which increases with increasing PTX amount, as the incorporated PTX decelerates polymer recrystallization [31,70]. Liggins and Burt explained it in a way that hydrogen bonding between PLLA and PTX lead to accumulation of PTX in the amorphous regions and therefore in a reduced rate of polymer chain diffusion and thus slowed crystallization [71]. In accordance to this, our results indicate a similar behavior for thin films accessible by spray coating. No PTX signal at around 217 °C was detected in the thermogram leading to the conclusion that PTX is completely blended into the polymer. However, the appearance of an exothermic peak at 104–107 °C for 20 *w*/*w*% PTX indicates that thermal annealing at 80 °C is not sufficient to achieve thermal equilibrium state and therefore may have an impact on long term properties in respect to biomedical application of such a drug containing matrix, e.g., shelf life.

For PLLA/SIR films, the presence of a SIR melting peak, up to 199.5 ± 0.6 °C, which is considerably higher than the *T_m_* of the pure SIR component at 183.9 ± 0.5 °C, leads to the conclusion that SIR is not completely miscible with PLLA and phase separation occurs. This is underlined by means of the inhomogenities observed in SEM imaging. A similar observation has been made by Nukula for SIR in poly (butyl methacrylate)/poly(ethylene vinyl acetate) (PBMA/PEVA) [72]. They assumed from the broaden appearance of the SIR melting peak close to the melting peak of pure SIR in the thermogram that the drug is mainly encapsulated in an amorphous state with only little crystallinity. It is still unclear whether the SIR crystal formation is an effect due to immiscibility of PLLA or appeared when generating the test samples as SIR showed limited solubility in CHCl_3_. To bypass this, the drug had been dissolved in MeOH, which has been added to the CHCl_3_-polymer-solution. Precipitation may have been occurred at this point leading to micro crystals, though this has not been investigated in this study.

In the case of DEX incorporation, the shift of the endothermic peak *T_m,DEX_* from 231.8 ± 1.5 °C to 240.1 ± 1.4 °C indicates an interaction between DEX and PLLA. Consequently, a certain amount of DEX must have been incorporated into the PLLA matrix up to a threshold whereas phase separation occurs. Otherwise, we observed similar behavior as it has been reported for DEX containing PCL nanofibers by means of a drug melting signal [73]. As Martins et al. concluded, DEX precipitates from the polymer/drug solution during the manufacturing process in a similar way as for SIR. Also, the increased *T_m_* of DEX leads to the conclusion that the drug exists in amorphous state in accumulated regions in the PLLA matrix. However, via Raman mapping, no DEX enriched areas could be detected. Both, for DEX and SIR, we assume that microcrystals have been formed which are below the resolution limit of the applied Raman mapping technique. As for area scans a 500 µm × 500 µm field with 50 dots per row and column was used, the resolution limit is ≥10 µm^2^. As for SIR, SEM indicated the formation of submicron crystals when incorporating DEX in PLLA.

CYCLO resulted in a slight increase in crystallinity of PLLA. The absence of a distinct *T_m,CYCLO_* signal in comparison to the drug *T_m_* for SIR or DEX showed complete blending of the drug with the polymer. 

Altogether, our results point out complex phase transitioning processes during manufacturing. As a drug-polymer-solvent system is present, interaction of the three components in solution as well as during solvent evaporation and thus shifting of solubilities must been taken into account for manufacturing and application as it may alter crucial parameters such as drug release or mechanical resilience. Still, real time analysis of such processes remains challenging and technically sophisticated. Further research, e.g., by means of X-ray diffraction, may help to understand the drug PLLA interaction in combination with solvent influences.

Mechanical analysis of PLLA films containing PTX gave seemingly contradictory results regarding DSC analysis, especially crystallinity of the PLLA matrix. In this case, a decrease in crystallinity of approx. 13%, from 37.8 ± 1.9% down to 33 ± 7%, was observed which is accompanied by an approximate doubling of the elongation at break, from 9.9 ± 0.7% to 23 ± 16% yet an increase in the elastic modulus of up to 25%, from 2250 ± 40 MPa to 3020 ± 100 MPa was observed. The latter indicates a higher embrittlement. SIR incorporation leads to higher elastic modulus values of about 34%, from 2250 ± 40 MPa to 3420 ± 220 MPa, indicating a hardening effect. However, elastic modulus values decrease as drug content increases and approximate at 20 *w*/*w*% SIR the original PLLA value. DSC data show an increase of crystallinity of PLLA with increasing SIR amount. This, in addition to the formation of SIR crystallites, counteracts the elastic modulus increasing influence of SIR molecules interacting with the polymer chains. Therefore, we assume that SIR shows complex behavior in the polymer crystal lattice. The investigation of such interactions of PTX or SIR with PLLA may be of certain interest for future work in particular by means of crystal structure analysis, as the formation of multiple phases crucially alters properties highly relevant for biomedical applications such as drug release. In addition, such effects are in particular important regarding dilation behavior and drug carrying coating integrity after the exposure of mechanical stress as it appears at stent implantation. 

DEX incorporation results in a tendency to lower elongation at break at high drug concentrations. Still, a decrease in elastic modulus of about 10%, from 3000 ± 200 MPa to 2690 ± 110 MPa, is observed. This may be due to the fact that on the one hand the drug interacts with the PLLA polymer chains on a molecular level, thus acting as plasticizer. However, on the other hand, the formation of aforementioned submicron crystals leads to the development of predetermined breaking points, therefore to a decrease in elongation at break of about 31%, from 8.8 ± 2.0% to 6.1 ± 0.9%. CYCLO shows with increasing drug ratio a reduction of elongation at break of ca. 40%, from 11 ± 6% to 6.4 ± 1.7% accompanied by an increase in elastic modulus and tensile strength of ca. 20%.

Regarding the mechanical analysis of PLLA/drug blends, it must be stated that handling of polymer films of a thickness of around 10 µm is challenging, as minimal defects or insufficient mounting in the testing device may drastically alter the results. However, the generation of thicker samples, e.g., by solvent casting process, may also be unsuitable as an increase in sample thickness leads to incomplete removal of residual solvent by evaporation. The remaining solvent may thus act as plasticizers, impairing the results on the drug influence. As for that, the literature and our DSC data for thin films obtained by spray-coating showed complete solvent removal after thermal annealing for which reason we decided to choose this manufacturing method for PLLA/drug films. Still, mechanical properties of such films, notably in the field of DES, are of particular interest. In the literature, some considerations about thermal analysis of drug incorporated polymers such as PLLA can be found. Yet, only limited data for mechanical investigations are available for reference. Consequently, further insight regarding the material properties of drug containing coatings is required.

## 5. Conclusions

Drug-eluting polymer coatings are an indispensable tool in biomedical device engineering, in particular for so-called combination products. Whereas drug release kinetics are clearly important and have been in the focus of extensive research, understanding the interaction of drugs with the drug-carrying polymer matrix is of great interest, especially in the field of drug-eluting stents. 

In this study, PLLA/drug thin films have been investigated regarding the influence of the drug on thermal and mechanical properties of the polymer matrix. SIR, PTX, CYCLO and DEX were blended with HMW PLLA in ratios of 10, 15, and 20 *w*/*w*% with respect to the polymer. Our results showed that the selected drugs lead to an increase in tensile strength of the coating material. Regarding thermal properties, SIR and DEX showed PLLA/drug phase separation. PTX and DEX incorporation resulted in thermal destabilization of the polymer, which is evident by the formation of exothermic peaks in the DSC thermograms. High amounts of PTX lead to a strong decrease in crystallinity of PLLA. CYCLO showed complete blending with the PLLA matrix and did only little affect the polymer properties.

Our aim was to contribute to the understanding of the complex interaction between PLLA and API. Different drugs possess crucially different chemical properties and ways of interacting when blended with polymers, and thus, the influence on the polymer matrix cannot be easily predicted. Advances in current established DES technology to overcome limitations and side effects, such as the risk of late stent thrombosis, inflammation and in stent-restenosis, demand for novel polymer/drug coatings. The present data underline that this in turn requires target-oriented thermal treatment to ensure shelf life and mechanical resilience.

## Figures and Tables

**Table 1:** Overview on selected commercially available DES (modified according to [25], drug loading data was taken from [27]).

Stent	Stent Material	Polymer	Absorption Time	Drug	Drug-Eluting Time	Manufacturer	Drug Loading
Cypher	Stainless steel	PEVA/PBMA	Permanent	Sirolimus	90 days	Cordis	140 μg/cm^2^
Taxus Express	Stainless steel	SIBS	Permanent	Paclitaxel	>180 days	Boston Scientific	100 μg/cm^2^
Xience Alpine	CoCr	PVDF- HFP	Permanent	Everolimus	120 days	Abbott Laboratories	100 μg/cm^2^
Resolute Integrity	CoNi with Pt-Ir	BioLinx	Permanent	Zotarolimus	180 days	Medtronic	-
Orsiro	CoCr	PLLA	15 months	Sirolimus	100–120 days	Biotronik	1.4 μg/mm^2^
Ultimaster	CoCr	PDLLA-PCL	3–4 months	Sirolimus	3–4 months	Terumo Interventional Systems	-
Synergy	PtCr	PLGA	3–4 months	Everolimus	3 months	Boston Scientific	38–179 μg/stent
Nobori	Stainless steel	PDLLA	6–9 months	Biolimus	6–9 months	Terumo	15.6 μg/mm^2^

PEVA = poly(ethylene-co-vinyl acetate); PBMA = poly(n-butyl methacrylate); SIBS = poly(styrene-*b*-isobutylene-*b*-styrene); PVDF-HFP = poly(vinylidene fluoride-co-hexafluoropropylene); BioLinx = blend of methacrylate and vinylpyrrolidone based polymers; PLLA = poly-l-lactide; PDLLA-PCL = poly(dl-lactide-co-caprolactone); PLGA = poly(lactic-co-glycolic acid); PDLLA = poly(dl-lactide).

**Table 2:** An overview concerning the drugs used in this study for blending with PLLA and their selected properties.

Drug	Structural Formula	Space Filling Model Illustration	PSA (in Å^2^) ^1^	logP	M_W_ (g/mol)
Paclitaxel (PTX)	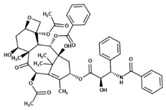	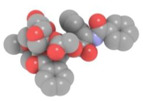	221	3 [18]	853.9
Sirolimus (SIR)	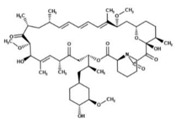	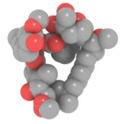	195	7.45 [55]	914.2
Dexamethasone (DEX)	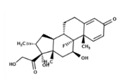	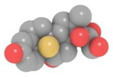	95	1.83 [56]	392.5
CYCLOsporine (CYCLO)	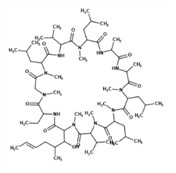	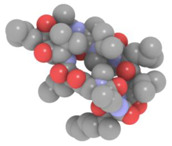	279	1.4 [57]	1202.6

^1^ Polar surface area (PSA) values were taken from PubChem and calculated via CACTVS [58], according to the information on the website. MarvinSketch was used for molecular structure drawing (Marvin v.20.11, 2020, ChemAxon, Budapest, Hungary). Space filling models were created with QuteMol (v. 0.4.1) [59] using pdb-files created from MarvinSketch.

**Table 3:** DSC results (glass transition (*T_g_*), melting temperature (*T_m_*) and degree of crystallinity (*χ*)) of PLLA/drug spray coated films in different ratios (90/10, 85/15, 80/20 *w*/*w*%) (n = 5 for each group). * in T_m,DEX*_ indicates that shift in melting point occurs in comparison to pure drug.

**PLLA/PTX**	***T_g_* (°C)**	**Δ*H_PLLA_* (J/g)**	***χ* (%)**	***T_m,PLLA_* (°C)**	**Δ*H_PTX_* (J/g)**	***T_m,PTX_* (°C)**
100/0	72.8 ± 0.5	35.4 ± 1.8	37.8 ± 1.9	177.08 ± 0.25	---	---
90/10	75.4 ± 0.4	31 ± 7	33 ± 7	174.72 ± 0.19	---	---
85/15	74.4 ± 2.6	26 ± 10	27 ± 10	173.84 ± 0.07	---	---
80/20	69.2 ± 0.8	10.6 ± 1.6	11.3 ± 1.7	173.78 ± 0.13	---	---
0/100	---	---	---	---	32.4 ± 1.2	213.48 ± 0.17
**PLLA/SIR**	***T_g_* (°C)**	**Δ*H_PLLA_* (J/g)**	***χ* (%)**	***T_m,PLLA_* (°C)**	**Δ*H_SIR_* (J/g)**	***T_m,SIR_* (°C)**
100/0	72.8 ± 0.5	35.4 ± 1.8	37.8 ± 1.9	177.08 ± 0.25	---	---
90/10	73.45 ± 0.24	30 ± 5	32 ± 6	175.5 ± 0.5	43 ± 5	199.5 ± 0.6
85/15	74.9 ± 0.7	38 ± 8	41 ± 9	174.82 ± 0.28	45 ± 13	198.6 ± 0.6
80/20	73.9 ± 0.5	48.5 ± 2.9	52 ± 3	174.32 ± 0.07	49 ± 13	196.5 ± 0.5
0/100	---	---	---	---	64.9 ± 2.8	183.9 ± 0.5
**PLLA/DEX**	***T_g_* (°C)**	**Δ*H_PLLA_* (J/g)**	***χ* (%)**	***T_m,PLLA_* (°C)**	**Δ*H_DEX_* (J/g)**	T_m,DEX*_ (°C)
100/0	72.8 ± 0.5	35.4 ± 1.8	37.8 ± 1.9	177.08 ± 0.25	---	---
90/10	74.3 ± 0.5	44.0 ± 2.4	46.9 ± 2.6	177.3 ± 0.3	1.9 ± 0.8	231.8 ± 1.5
85/15	71.7 ± 1.7	38 ± 6	40 ± 6	177.0 ± 0.5	19 ± 4	238.2 ± 0.9
80/20	74.9 ± 0.5	42 ± 10	45 ± 11	177.69 ± 0.15	8 ± 5	240.1 ± 1.4
0/100	---	---	---	---	15.2 ± 2.8	263.5 ± 0.8
**PLLA/CYCLO**	***T_g_* (°C)**	**Δ*H_PLLA_* (J/g)**	***χ* (%)**	***T_m,PLLA_* (°C)**	**Δ*H_CYCLO_* (J/g)**	***T_m,CYCLO_* (°C)**
100/0	72.8 ± 0.5	35.4 ± 1.8	37.8 ± 1.9	177.08 ± 0.25	---	---
90/10	74.5 ± 0.7	43.1 ± 2.8	46 ± 3	174.51 ± 0.21	---	---
85/15	75.9 ± 2.0	40 ± 4	42 ± 4	174.61 ± 0.02	---	---
80/20	72.7 ± 2.4	36 ± 5	38 ± 6	173.7 ± 0.7	---	---
0/100	---	---	---	---	---	146.9 ± 0.7

**Table 4:** Mechanical properties (elastic modulus (*E*), tensile strength (*σ_max_*), elongation at break (*ε_B_*)), determined from uniaxial tensile tests of PLLA/drug spray coated films in different drug ratios (90/10, 85/15 and 80/20 *w*/*w*%).

**PLLA/PTX**	***E* (MPa)**	***σ_max_* (MPa)**	**ε_B_ (%)**
100/0	2250 ± 40	107.1 ± 1.3	9.9 ± 0.7
90/10	3020 ± 100	134 ± 4	23 ± 16
85/15	2980 ± 170	130 ± 16	16.1 ± 1.2
80/20	3320 ± 120	140 ± 5	12.3 ± 1.1
**PLLA/SIR**	***E* (MPa)**	***σ_max_* (MPa)**	**ε_B_ (%)**
100/0	2250 ± 40	107.1 ± 1.3	9.9 ± 0.7
90/10	3420 ± 220	156 ± 16	7.40 ± 0.17
85/15	3200 ± 700	160 ± 30	8.0 ± 0.7
80/20	2000 ± 220	115 ± 30	8.9 ± 0.8
**PLLA/DEX**	***E* (MPa)**	***σ_max_* (MPa)**	**ε_B_ (%)**
100/0	2250 ± 40	107.1 ± 1.3	9.9 ± 0.7
90/10	3000 ± 200	141 ± 9	8.8 ± 2.0
85/15	2940 ± 130	118 ± 8	11 ± 3
80/20	2690 ± 110	115 ± 2.4	6.1 ± 0.9
**PLLA/CYCLO**	***E* (MPa)**	***σ_max_* (MPa)**	**ε_B_ (%)**
100/0	2250 ± 40	107.1 ± 1.3	9.9 ± 0.7
90/10	2580 ± 240	119.2 ± 2.0	11 ± 6
85/15	2560 ± 110	107 ± 6	5.4 ± 0.7
80/20	2870 ± 190	141 ± 5	6.4 ± 1.7

## Data Availability

The data presented in this study are available upon request from the corresponding author.

## Supplementary Materials

The following are available online at https://www.mdpi.com/2073-4360/13/2/292/s1, Figure S1: Raman spectra of PLLA and PLLA with incorporated drugs at a ratio of drug/PLLA = 10/90 *w*/*w*%. Figure S2: IR spectra of PLLA and PLLA with incorporated cyclosporine A at a ratio of CYCLO/PLLA = 10/90 *w*/*w*%. Figure S3: Macrophotographical images of polymer film cutouts of PLLA and drug loaded PLLA films. Figure S4: SEM images of pure PLLA film drug and loaded PLLA films obtained via spray-coating. Figure S5: Raman mapping of PLLA with incorporated drugs at given drug/PLLA ratios. Mapping has been performed for the drug signal intensity according to the signal in the related Raman spectra. Figure S6: Representative contact angle images for PLLA reference and SIR/PLLA = 10/90 *w*/*w*%. Given are images of the droplets of deionized water for CA determination and images of MeI_2_ droplets for SFE calculation. Figure S7: Stress-strain curves of drug loaded PLLA films obtained via spray-coating.
