# Supplementary material for: Influence of Drug Incorporation on the Physico-Chemical Properties of Poly(l-Lactide) Implant Coating Matrices—A Systematic Study
Source: Polymers (Basel). 2021 Jan 18;13(2):292. doi: 10.3390/polym13020292 (PMC7831498; doi:10.3390/polym13020292)
Supplement: Supplementary file 1 [file polymers-13-00292-s001.pdf]

Supplement data:

Figure S1: Raman spectra of PLLA and PLLA with incorporated drugs at a ratio of drug/PLLA = 10/90 w/w%

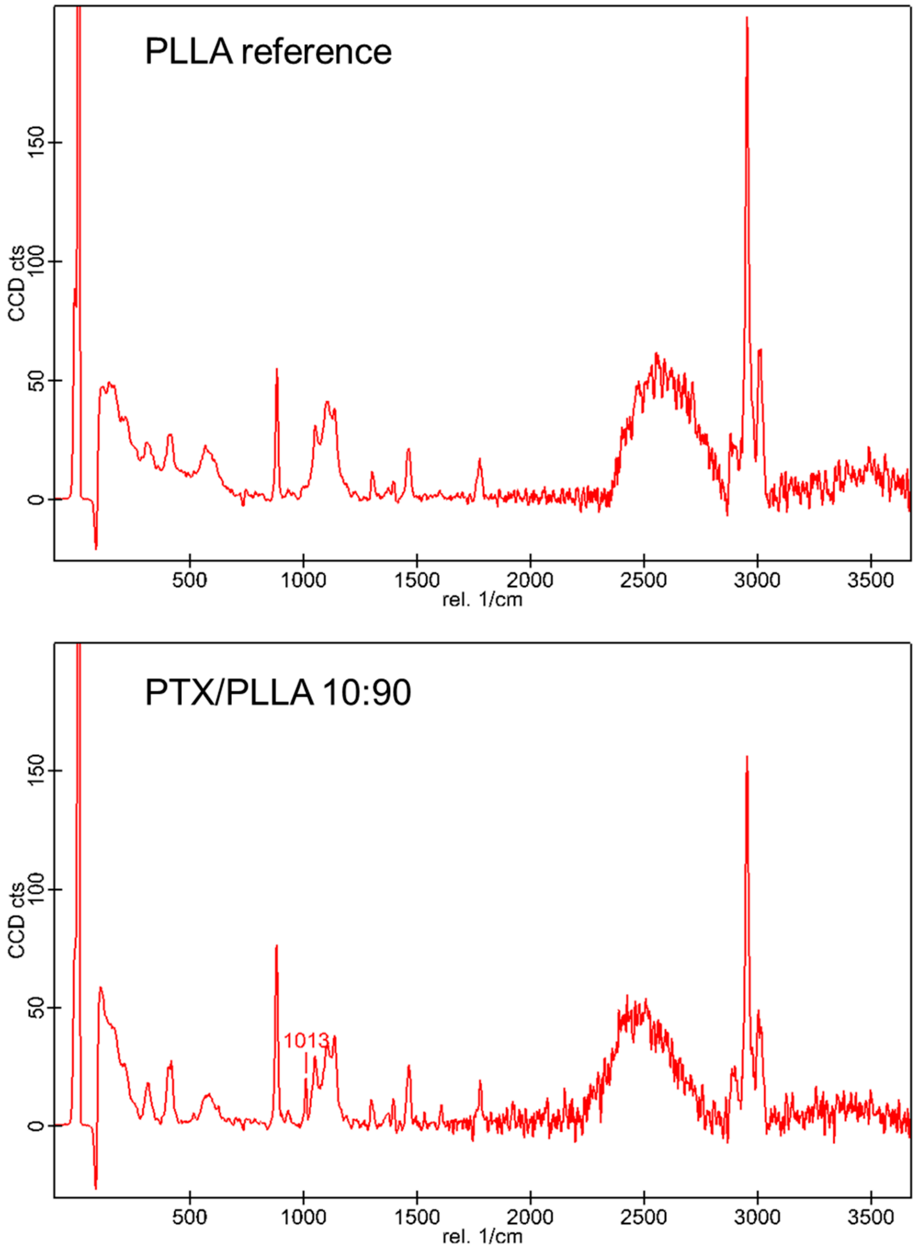

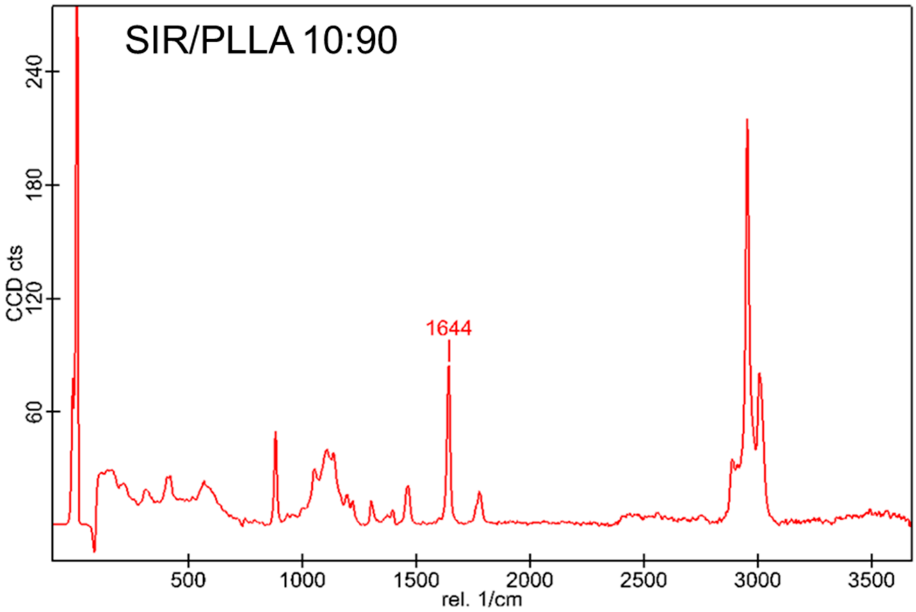
SIR/PLLA 10:90
CCD cts
240
180
120
60
1644
500
1000
1500
2000
2500
3000
3500
rel. 1/cm

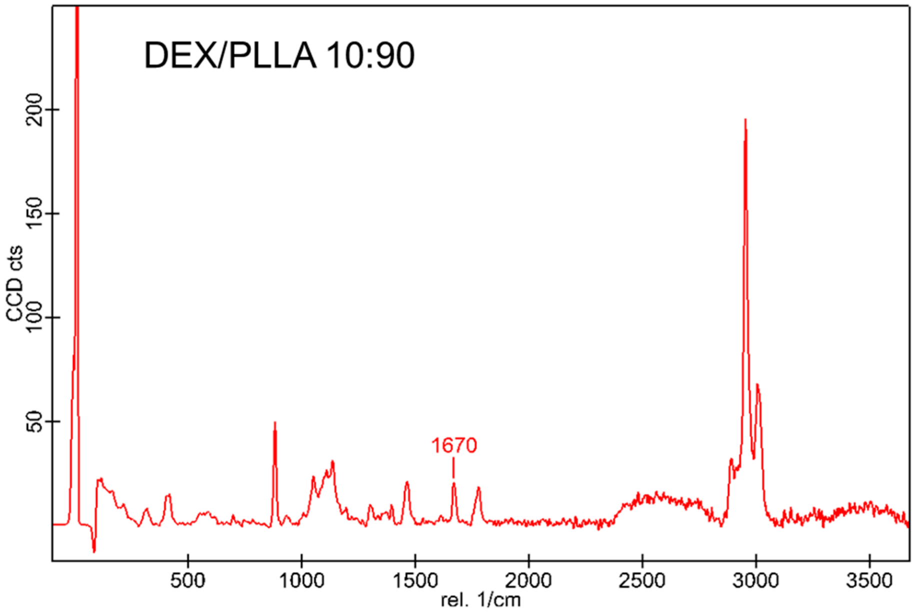
DEX/PLLA 10:90
CCD cts
200
150
100
50
1670
500
1000
1500
2000
2500
3000
3500
rel. 1/cm

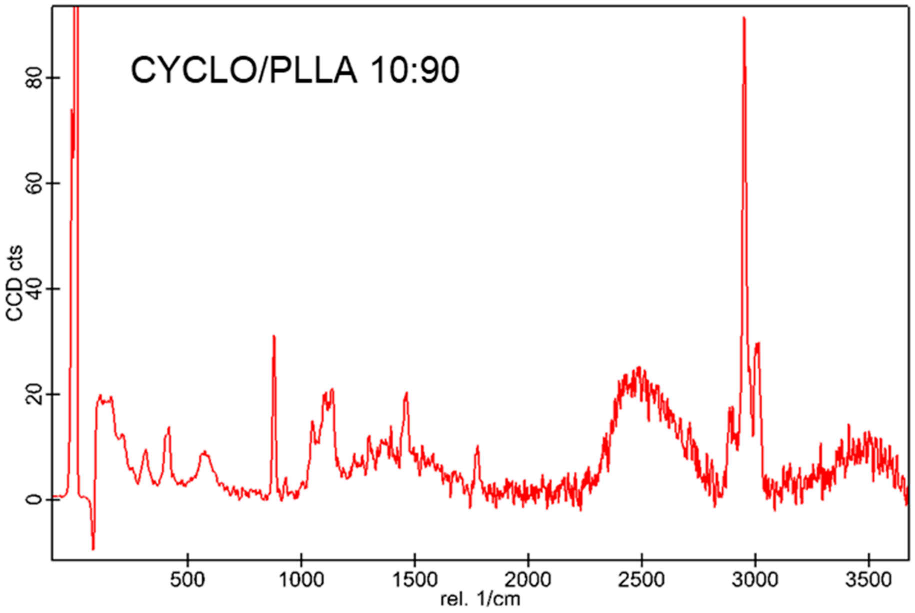
CYCLO/PLLA 10:90
CCD cts
80
60
40
20
0
500
1000
1500
2000
2500
3000
3500
rel. 1/cm

Figure S2: IR spectra of PLLA and PLLA with incorporated cyclosporine A at a ratio of CYCLO/PLLA = 10/90 w/w% with assigned bands for determination of drug incorporation.

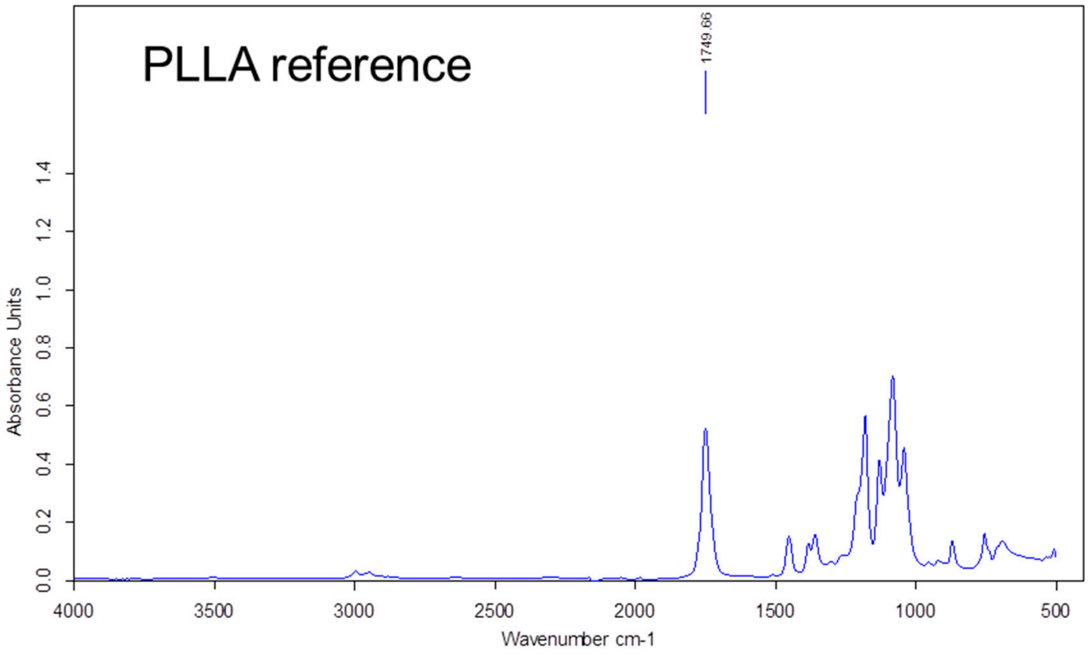

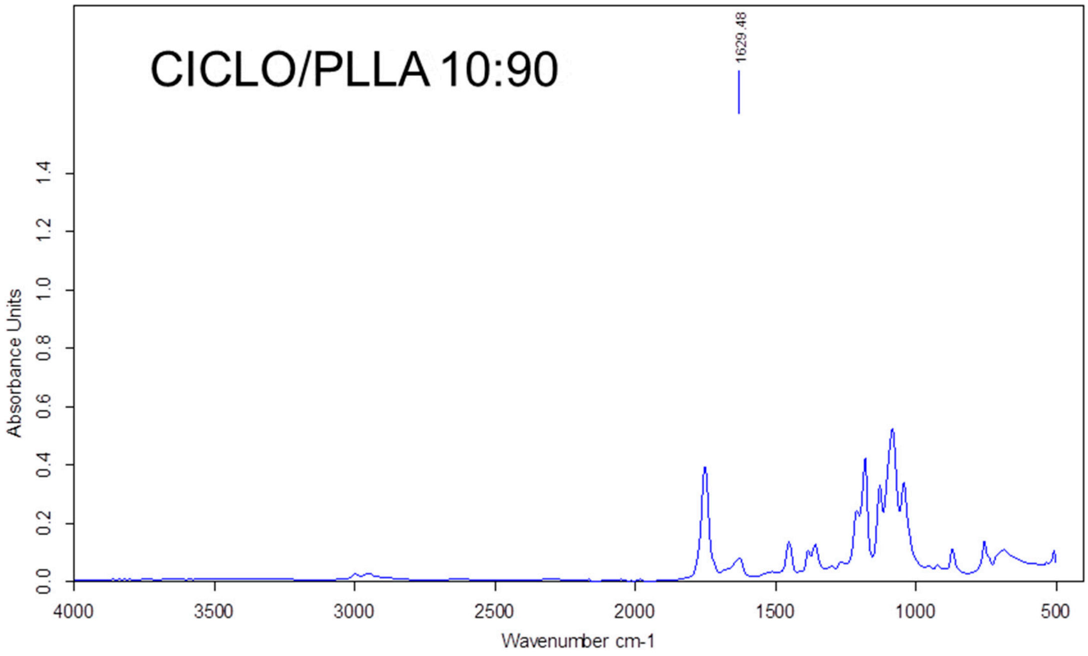

Figure S2.1: IR spectra of PLLA and PLLA with incorporated sirolimus at 10, 15 and 20 w/w% and sirolimus reference spectrum.

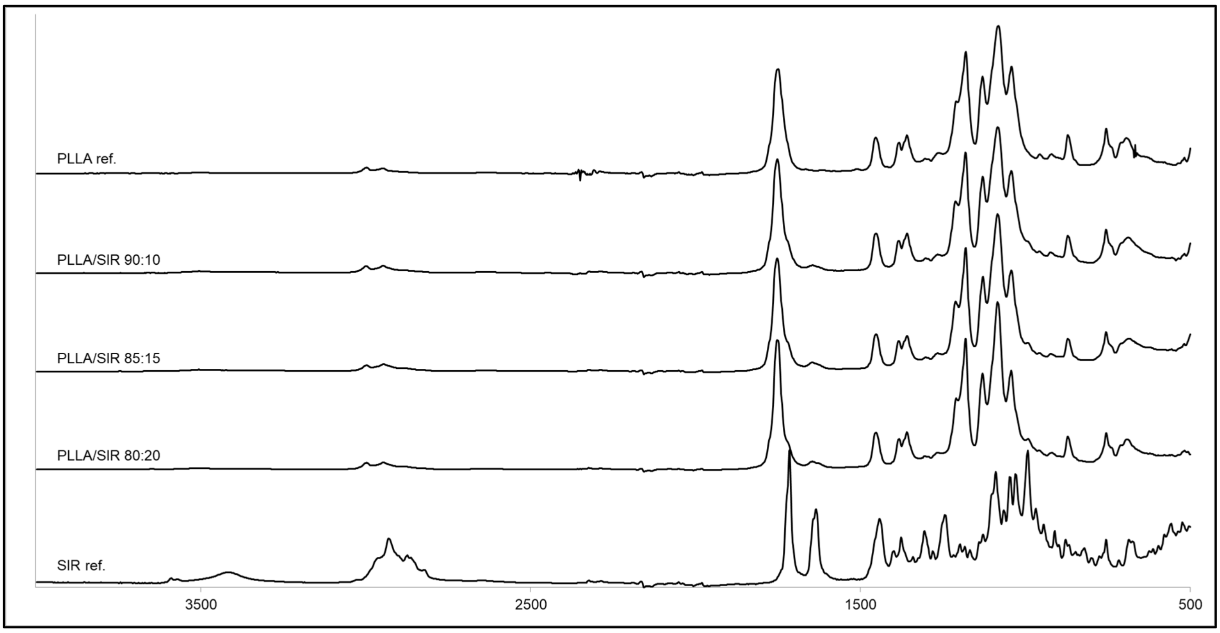

Figure S2.2: IR spectra of PLLA and PLLA with incorporated paclitaxel at 10, 15 and 20 w/w% and paclitaxel reference spectrum.

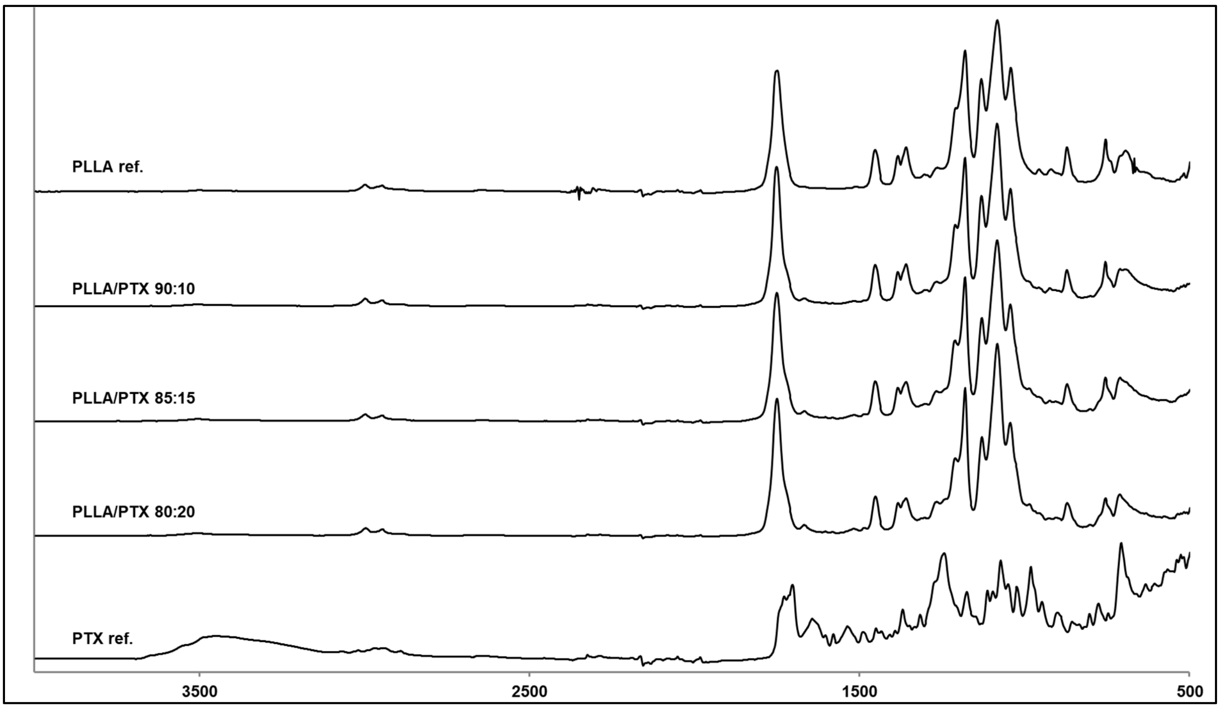

Figure S2.3: IR spectra of PLLA and PLLA with incorporated dexamethasone at 10, 15 and 20 w/w% and dexamethasone reference spectrum.

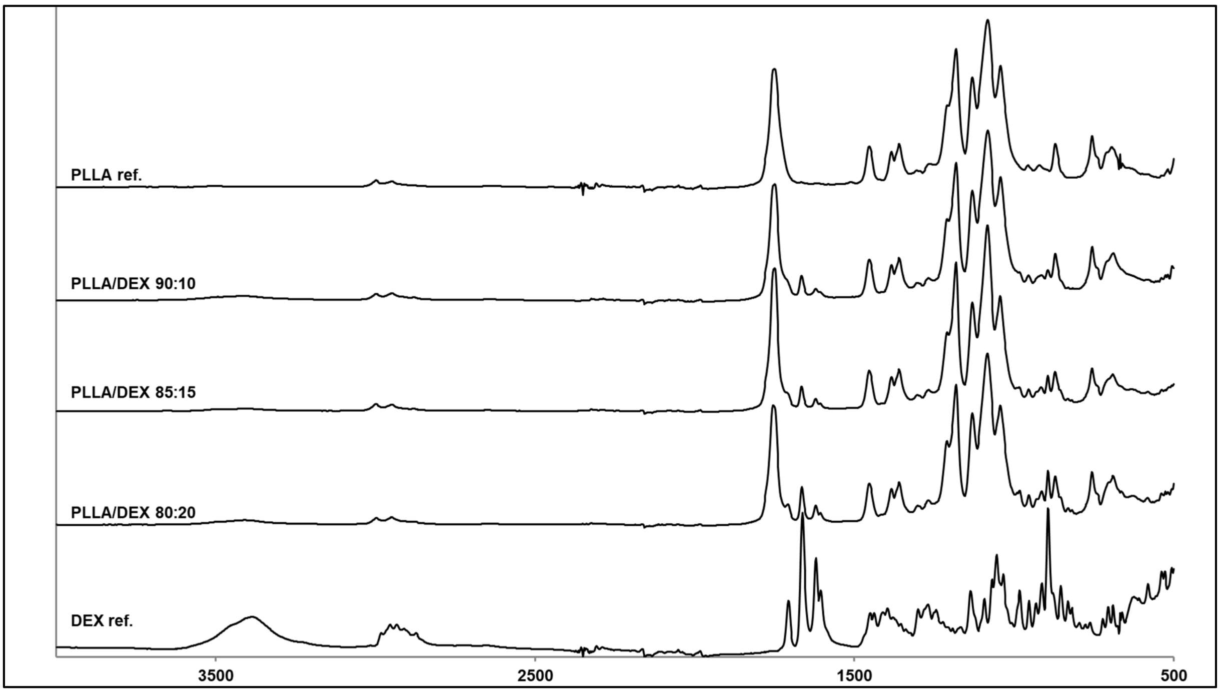

Figure S2.4: IR spectra of PLLA and PLLA with incorporated cyclosporine A at 10, 15 and 20 w/w% and cyclosporine A reference spectrum.

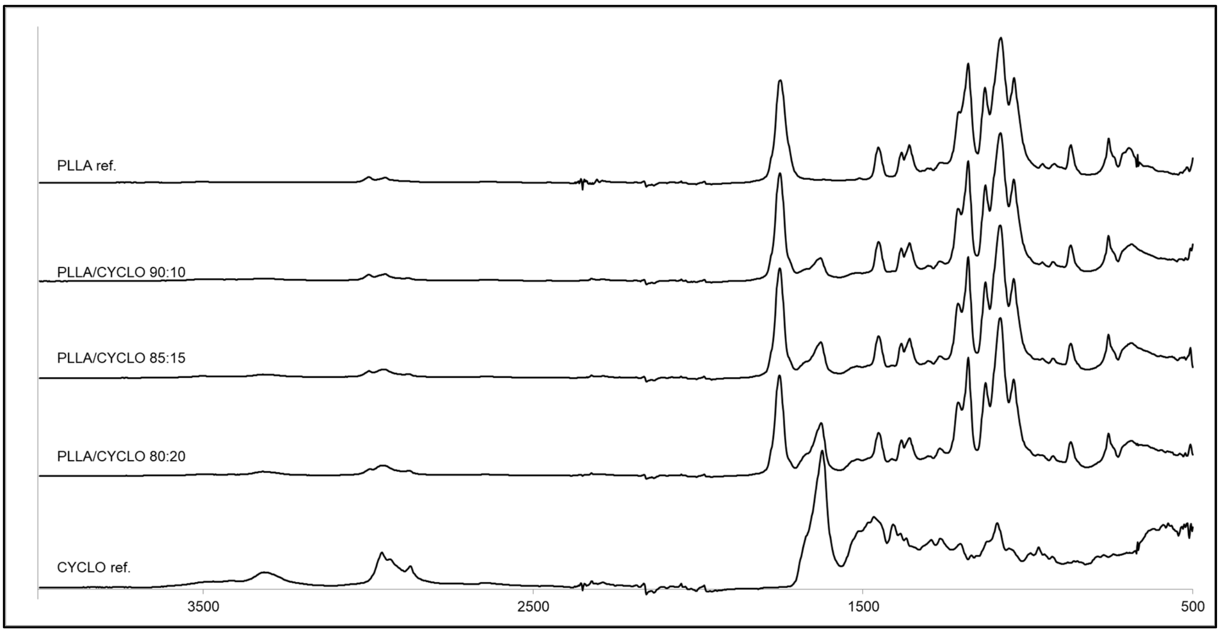

Figure S3: Macrophotographical images of polymer film cutouts of PLLA and drug loaded PLLA films.

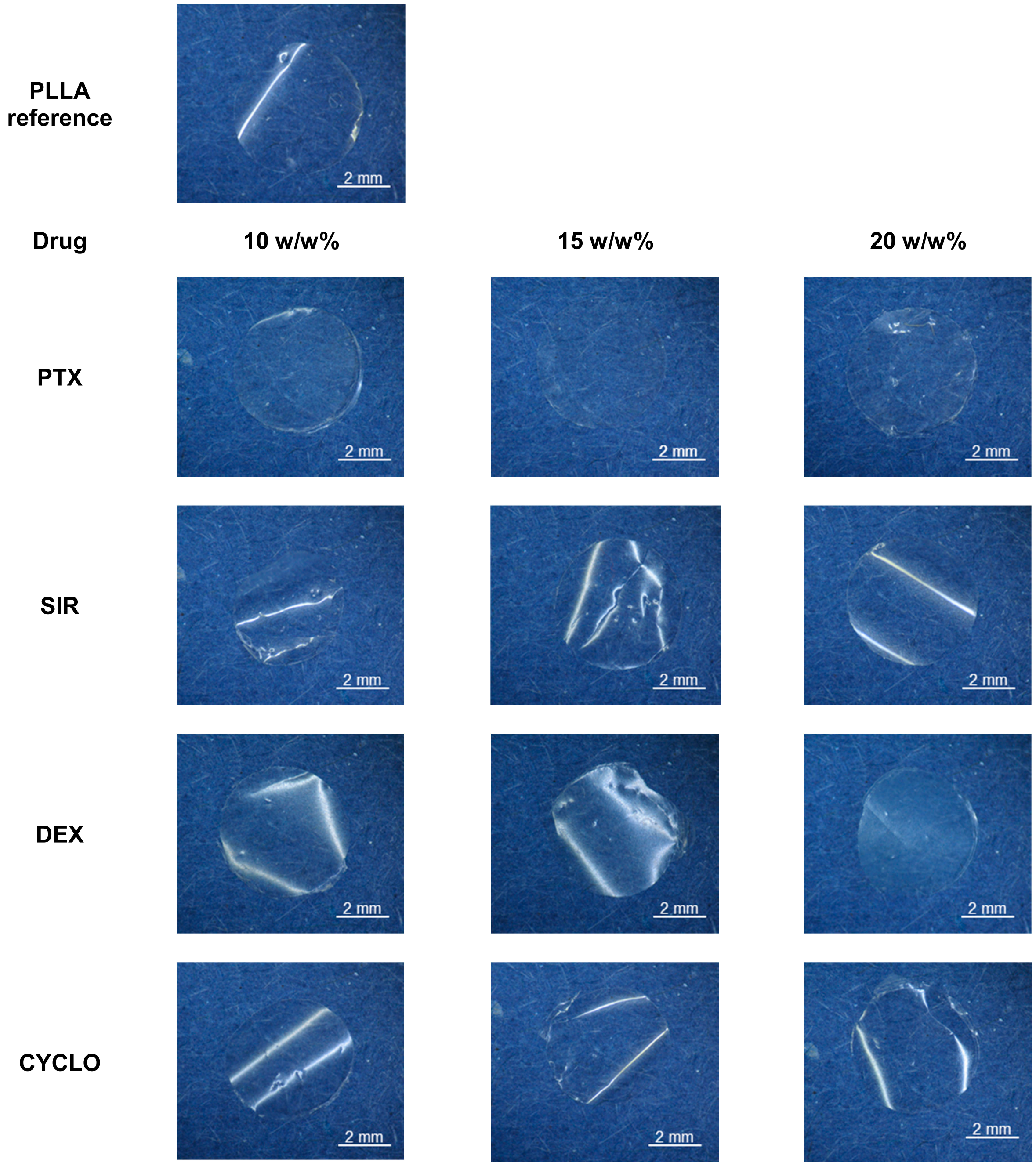

Figure S4: SEM images of pure PLLA film drug and loaded PLLA films obtained via spray-coating.

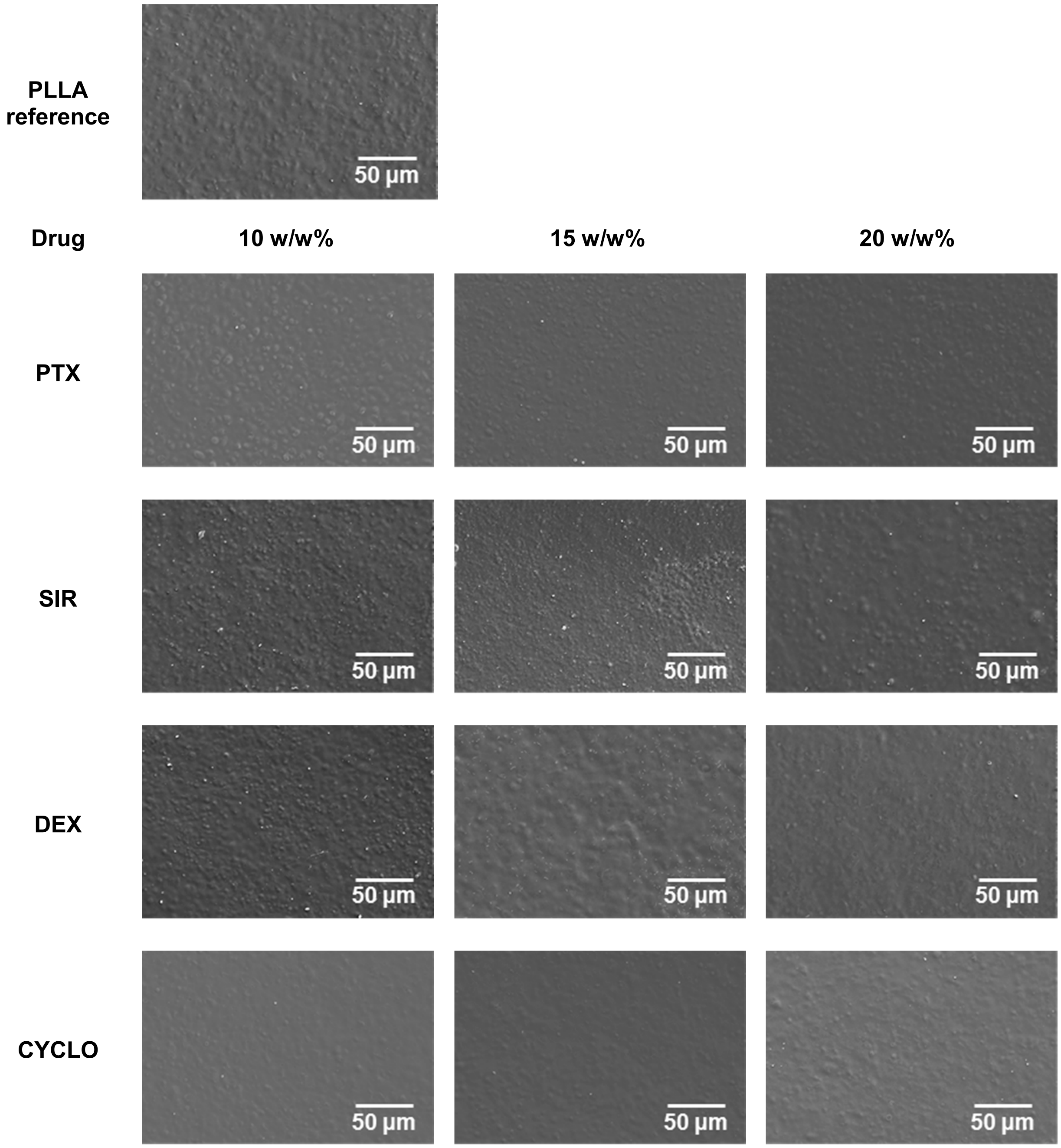

Figure S5: Raman mapping of PLLA with incorporated drugs at given drug/PLLA ratios. Mapping has been performed for the drug signal intensity according to the signal in the related Raman spectra.

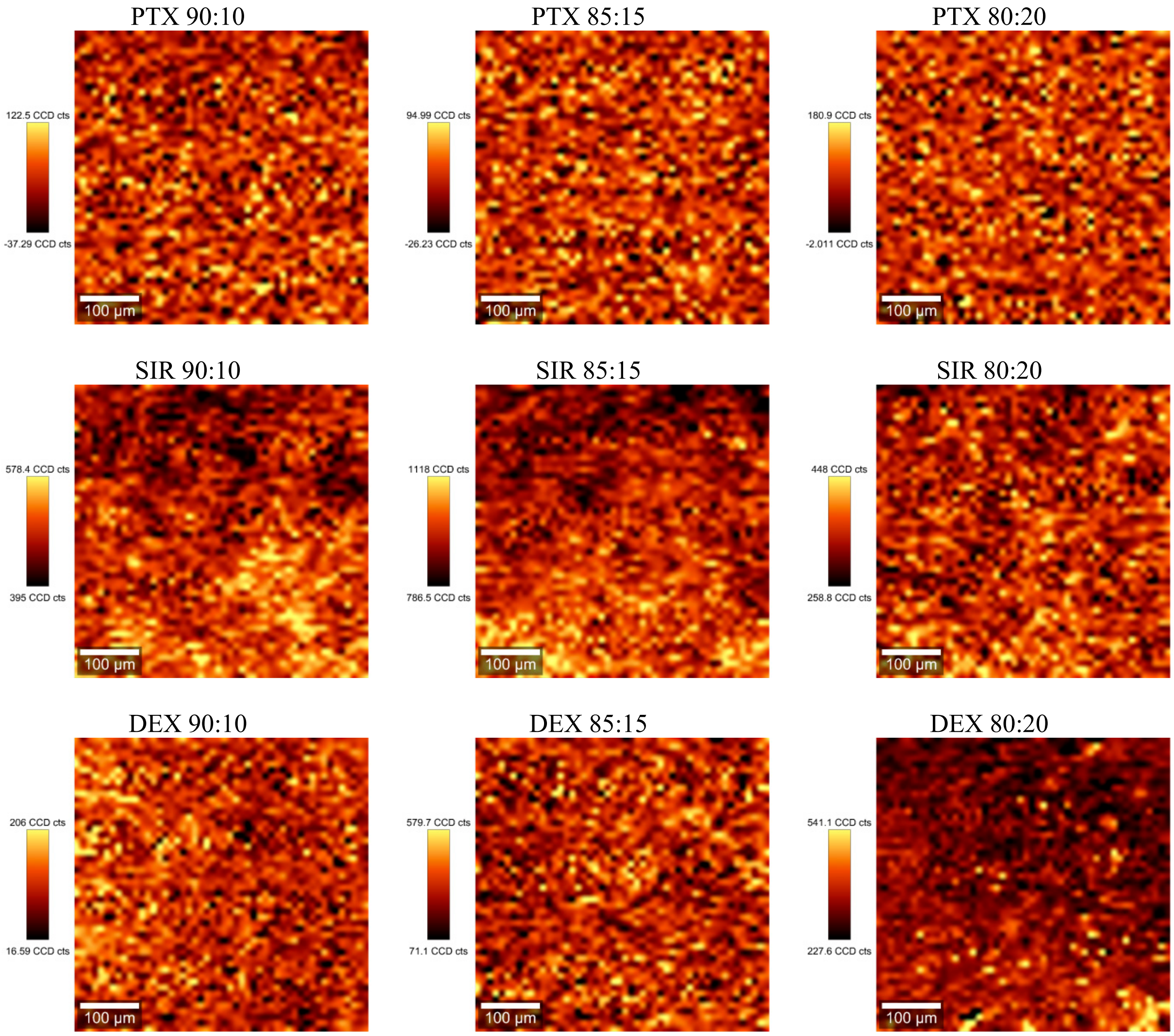

Figure S6: Representative contact angle images for PLLA reference and SIR/PLLA = 10/90 w/w%. Given are images of the droplets of deionized water for CA determination and images of MeI_2 droplets for SFE calculation.

PLLA reference CA H_2O

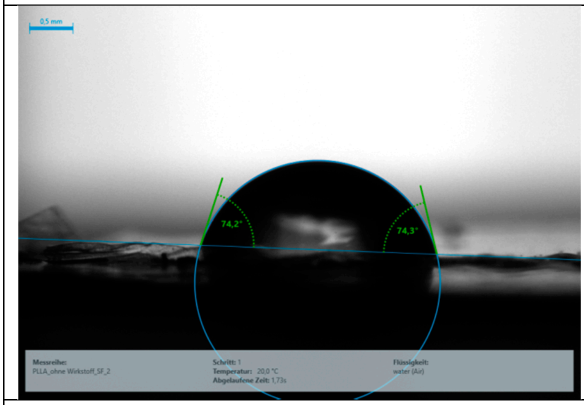

PLLA reference CA MeI_2

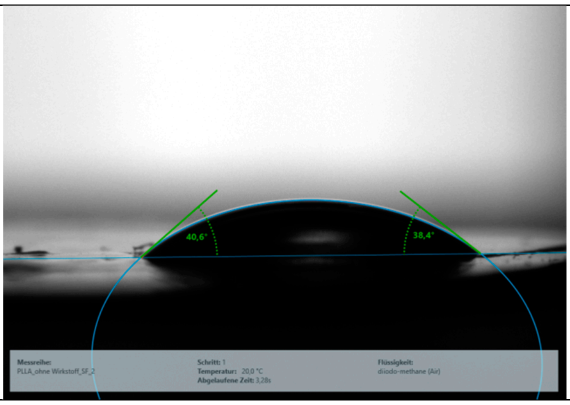

PLLA/SIR 85:15e CA H_2O

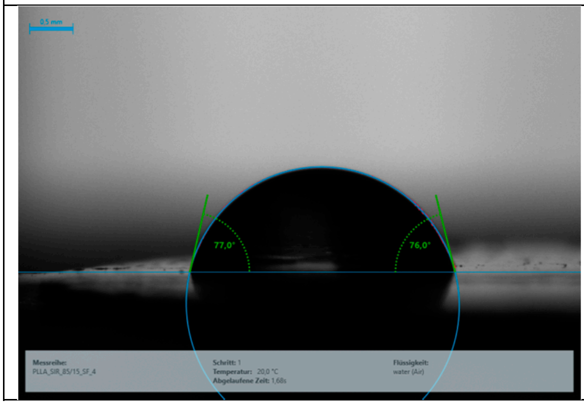

PLLA/SIR 85:15 CA MeI_2

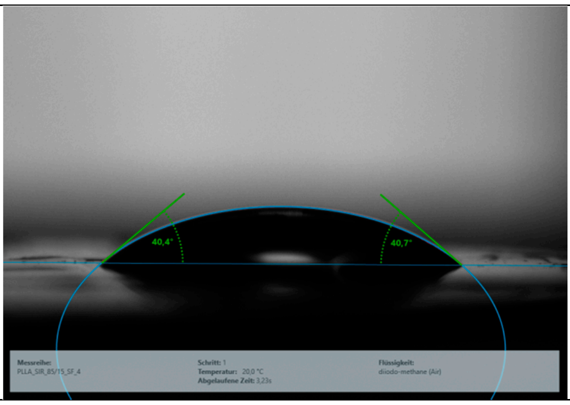

Figure S7: Stress-strain curves of drug loaded PLLA films obtained via spray-coating.

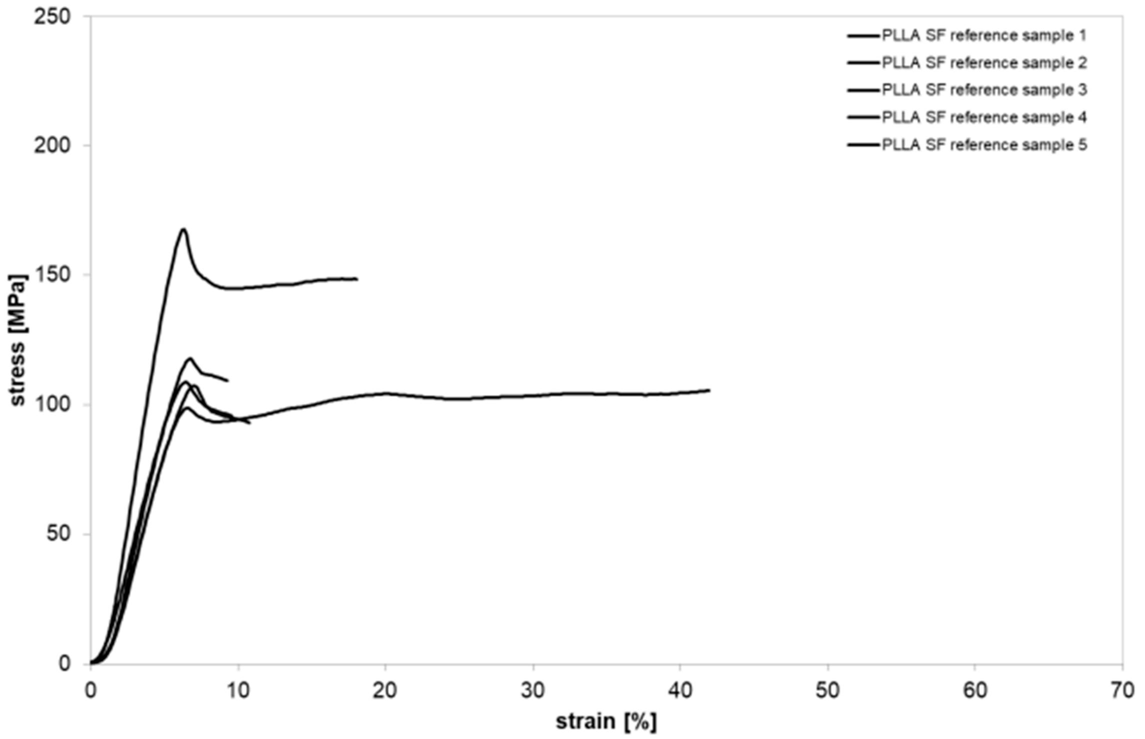

Figure S7.1: Stress strain curves of spray-coated PLLA film without any drug content.

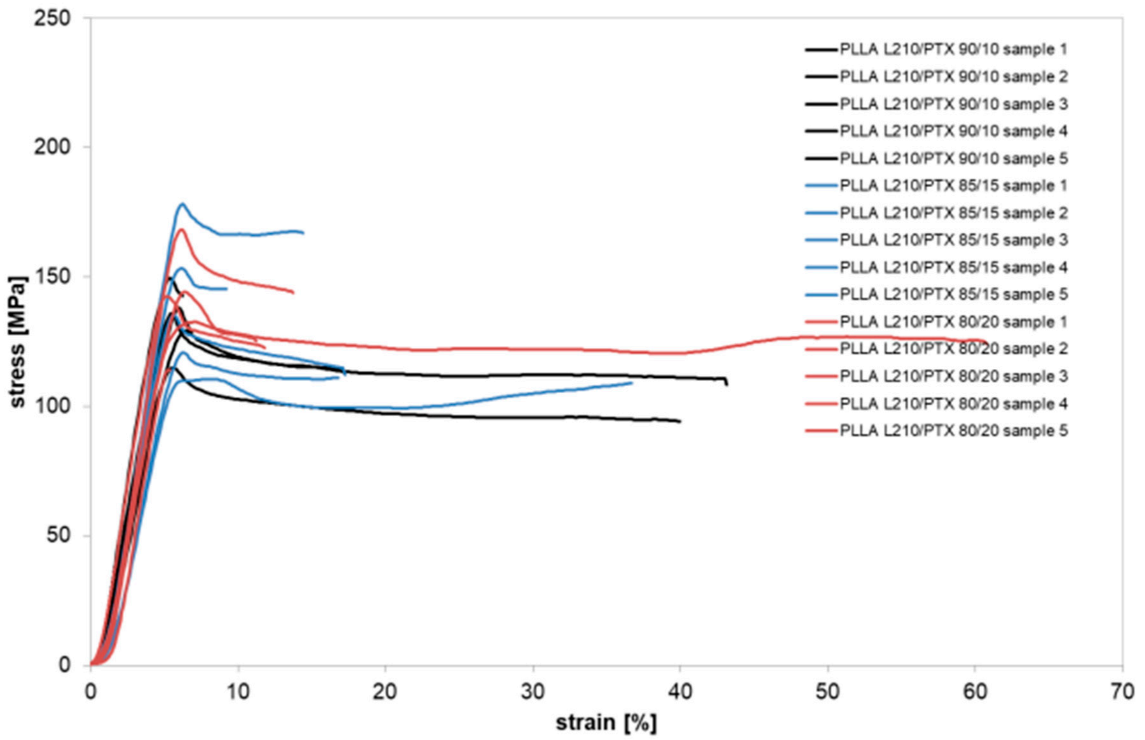

Figure S7.2: Stress strain curves of spray-coated PLLA/PTX film.

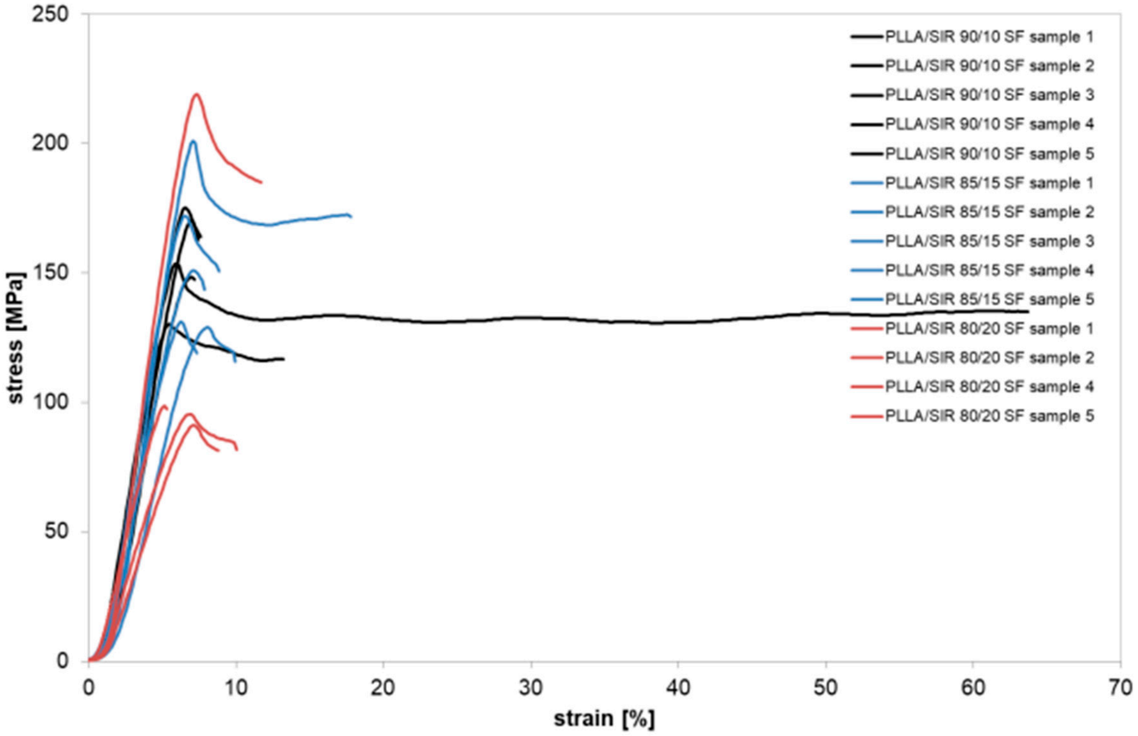

Figure S7.3: Stress strain curves of spray-coated PLLA/SIR film.

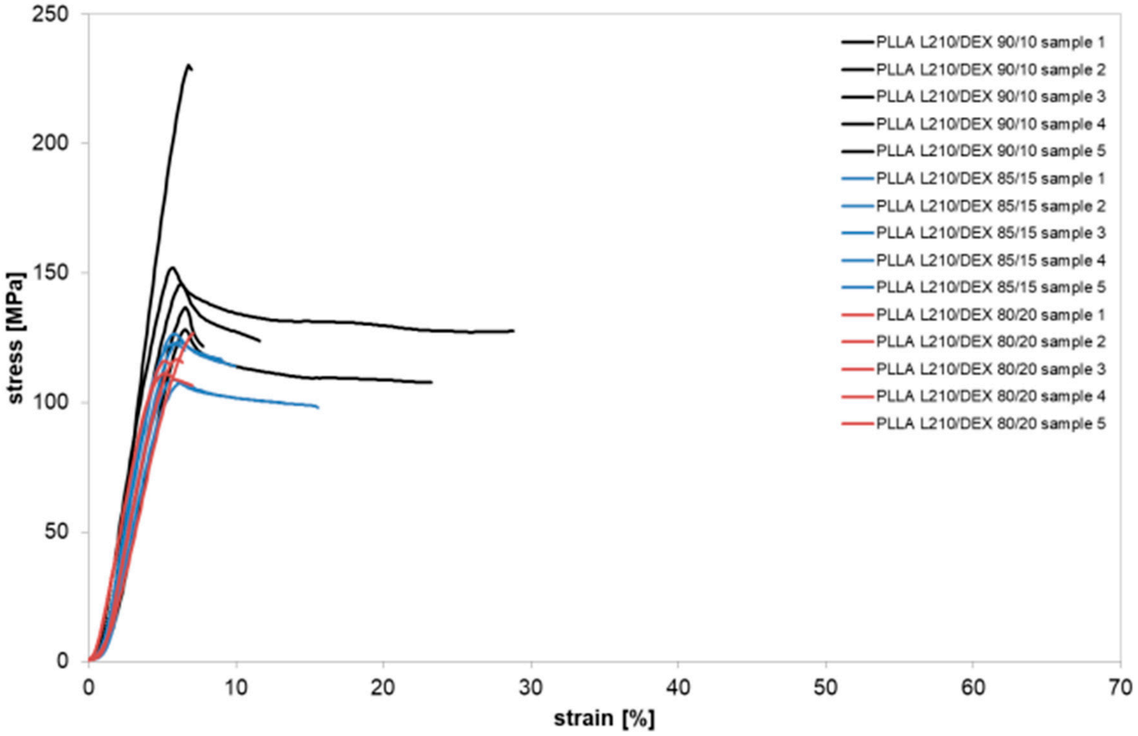

Figure S7.4: Stress strain curves of spray-coated PLLA/DEX film.

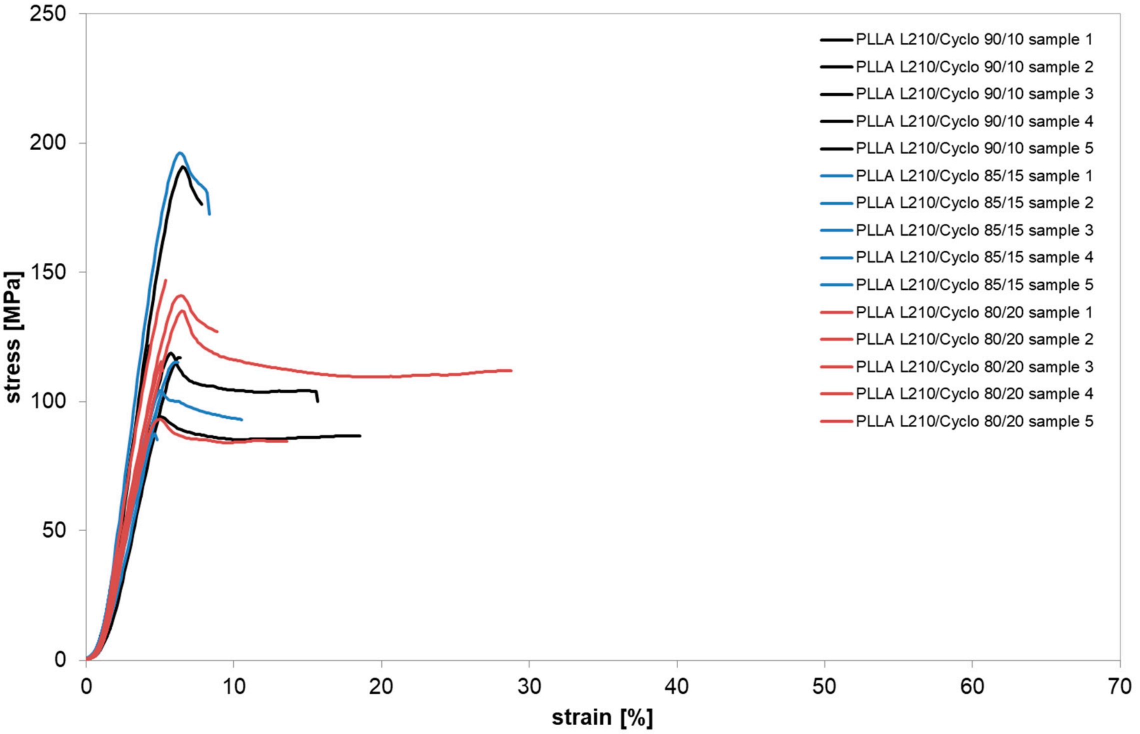

Figure S7.5: Stress strain curves of spray-coated PLLA/CICLO film.
